# Mitoxantrone targets both host and bacteria to overcome vancomycin resistance in *Enterococcus faecalis*

**DOI:** 10.1126/sciadv.add9280

**Published:** 2023-02-22

**Authors:** Ronni A. G. da Silva, Jun Jie Wong, Haris Antypas, Pei Yi Choo, Karlyn Goh, Shreya Jolly, Cui Liang, Leona Tay Kwan Sing, Mark Veleba, Guangan Hu, Jianzhu Chen, Kimberly A. Kline

**Affiliations:** ^1^ Singapore-MIT Alliance for Research and Technology, Antimicrobial Drug Resistance Interdisciplinary Research Group, Singapore, Singapore.; ^2^ Singapore Centre for Environmental Life Sciences Engineering, Nanyang Technological University, Singapore, Singapore.; ^3^ Interdisciplinary Graduate Programme, Nanyang Technological University, Singapore, Singapore.; ^4^ School of Biological Sciences, Nanyang Technological University, Singapore, Singapore.; ^5^ Koch Institute for Integrative Cancer Research and Department of Biology, Massachusetts Institute of Technology, Cambridge, MA, USA.; ^6^ Department of Microbiology and Molecular Medicine, Faculty of Medicine, University of Geneva, Geneva, Switzerland.

## Abstract

Antibiotic resistance critically limits treatment options for infection caused by opportunistic pathogens such as enterococci. Here, we investigate the antibiotic and immunological activity of the anticancer agent mitoxantrone (MTX) in vitro and in vivo against vancomycin-resistant *Enterococcus faecalis* (VRE). We show that, in vitro, MTX is a potent antibiotic against Gram-positive bacteria through induction of reactive oxygen species and DNA damage. MTX also synergizes with vancomycin against VRE, rendering the resistant strains more permeable to MTX. In a murine wound infection model, single-dose MTX treatment effectively reduces VRE numbers, with further reduction when combined with vancomycin. Multiple MTX treatments accelerate wound closure. MTX also promotes macrophage recruitment and proinflammatory cytokine induction at the wound site and augments intracellular bacterial killing in macrophages by up-regulating the expression of lysosomal enzymes. These results show that MTX represents a promising bacterium- and host-targeted therapeutic for overcoming vancomycin resistance.

## Teaser

Mitoxantrone represents a promising dual bacterium- and host-targeted therapeutic for overcoming vancomycin resistance.

## INTRODUCTION

Antibiotic resistance represents a major global health threat. Recent estimates attribute 4.95 million deaths in 2019 to antimicrobial resistance (AMR) (*1*), with this number predicted to climb to 10 million deaths annually by 2050 (*2*). Thus, multipronged treatment approaches, including antimicrobials that overcome existing resistance mechanisms and host-directed adjuvant therapies that enhance natural immune responses, are emerging as important alternatives to fight bacterial infections (*3*). *Enterococcus faecalis, Staphylococcus aureus,* and *Pseudomonas* are among the most frequently isolated bacterial species from wounds including burns, diabetic foot ulcers, surgical sites, and chronic wounds (*4*–*7*). *E. faecalis* have low-level intrinsic resistance to penicillin, ampicillin, and cephalosporins (*8*). An estimated ~30% of health care–associated enterococcal infections are caused by vancomycin-resistant strains, further reducing treatment options (*9*). Vancomycin-resistant enterococci (VRE) are on the Centers for Disease Control and Prevention (CDC) serious threat watch list, with $539 million in estimated attributable health care costs in 2017 alone (*9*). This public health threat will continue to grow as enterococci acquire resistance to last resort antimicrobials such as quinupristin-dalfopristin, linezolid, daptomycin, and tigecycline (*8*, *10*).

Understanding the molecular mechanism behind resistance is fundamental to develop new strategies to fight it. Vancomycin inhibits Gram-positive bacteria by targeting cell wall synthesis (*11*). In vancomycin-resistant strains, proteins of a two-component regulatory system (such as VanRS) sense the binding of vancomycin to peptidoglycan (PG) precursor d-alanyl-d-alanine (d-Ala-d-Ala) dipeptide termini. Cell wall disturbances trigger the PG precursor replacement to d-alanyl-d-lactate (d-Ala-d-Lac) by other proteins encoded by the *van* operon, impeding vancomycin binding. The d,d-carboxypeptidase VanY removes the terminal d-Ala residue from PG in the cell wall, and the enzyme VanX hydrolyzes d-Ala-d-Ala, thereby reducing the pool of available d-Ala-d-Ala (*10*, *12*) that vancomycin could bind to.

In addition to intrinsic and acquired AMR that complicate the treatment of enterococcal infection, we have previously shown that extracellular *E. faecalis* can subvert immune activation during infection (*13*). Moreover, *E. faecalis* can be internalized by keratinocytes and macrophages, alter endolysosomal trafficking, and replicate intracellularly, leading to a hyper-infective phenotype (*14*). Furthermore, persistent *E. faecalis* wound infection is associated with lowered cytokine levels in the wound, which could impair wound healing (*15*). Thus, infections with *E. faecalis* are perfectly positioned to benefit from host-targeted immunotherapies that may counteract the immunomodulating action of this microbe.

One potential target for host-directed immunotherapy is to promote enterococcal clearance by macrophages. Following phagocytosis, macrophages generate reactive oxygen and nitrogen species (*16*, *17*), mobilize transition metals to intoxicate microbial cells (*18*), and acidify the phagosome to activate lysosomal enzymes to eliminate internalized bacteria (*19*). Macrophages also present pathogen antigens to other immune cells and secrete cytokines to recruit immune cells to the site of infection (*20*). Moreover, macrophages have high transcriptional plasticity, which allow them to polarize and change their phenotypic profile depending on host or external stimuli (*21*, *22*). Macrophage polarization can exist on a spectrum, including macrophages associated with a proinflammatory state in response to infection and macrophages linked to tissue repair and remodeling (*23*). Together, because of their plasticity and pivotal role in fighting infections and promoting tissue repair, drugs that can reprogram macrophages toward an optimal response may improve infection outcomes.

Repurposed compounds offer an excellent opportunity in the pursuit of new therapies that can target both the host and the pathogen. The vast knowledge and safety validation of drugs deployed to treat other health conditions can markedly reduce the time and cost in the development of new therapeutical approaches (*24*). From a pool of 4126 compounds (including 760 U.S. Food and Drug Administration–approved drugs), we previously identified a series of compounds as capable of reprogramming macrophages into a proinflammatory state or anti-inflammatory state (*25*). In this study, we evaluated selected compounds for antibiotic activity and macrophage augmentation, leading to enhanced bacterial clearance. Here, we report that mitoxantrone (MTX), an antineoplastic agent commonly used to treat acute leukemia, prostate and breast cancers, as well as multiple sclerosis by disrupting DNA replication in mammalian cells, is both antimicrobial and immunomodulatory. Our data show that MTX both resensitizes VRE to killing by vancomycin and promotes macrophage recruitment and activation, rendering MTX an attractive candidate for difficult-to-treat VRE infections.

## RESULTS

### MTX exhibits potent antibiotic activities in vitro and in vivo

We evaluated 18 compounds that were previously shown to reprogram macrophages (*25*) for their ability to enhance macrophage-mediated killing of intracellular bacteria in vitro. MTX was among the most potent compounds (fig. S1 and table S1), and we therefore tested its antibiotic activity by measuring the minimal inhibitory concentration (MIC) against a panel of both Gram-positive and Gram-negative bacterial strains in the absence of macrophages. Bacteria were grown in increasing concentrations of MTX, ranging from 0.4 to 51.2 μg/ml, for 24 hours. MTX was 10- to 20-fold more potent in inhibiting the growth of Gram-positive bacteria (~1 μg/ml of MIC) compared to Gram-negative bacteria (Table 1). Among the Gram-positive bacteria, MTX similarly inhibited the growth of vancomycin-resistant *E. faecalis* V583 (our prototypic VRE strain going forward), *Enterococcus faecium* AUS0004 and *E. faecium* E745, vancomycin-sensitive *E. faecalis* OG1RF, *S. aureus* USA300 [methicillin-resistant *S. aureus* (MRSA)], and seven other vancomycin-sensitive enterococcal and staphylococcal strains.

**Table 1.** MTX is more efficient against Gram-positive bacteria in DMEM. MTX MIC for different bacterial species.

Gram-negative	MIC (μg/ml)	Gram-positive	MIC (μg/ml)
*E. coli* UTI89	6.45	*E. faecalis* V583	1.615
*E. coli* MG1655	25.87	*E. faecium* AUS0004	0.808
*E. coli* EC958	25.87	*E. faecium* E745	1.615
*P. aeruginosa* PAO1	12.94	*E. faecalis* OG1RF	0.808
*S. Typhimurium* 14028S	25.87	*S. aureus* USA300	0.808
–	–	*E. faecalis* 12030	1.560
–	–	*E. faecalis* TTSHW-EF43	1.560
–	–	*E. faecalis* ATCC 29121	1.560
–	–	*S. aureus* 8325-4	0.780
–	–	*S. aureus* MN8	1.560
–	–	*S. aureus* 15981	0.780
–	–	*S. aureus* ISP479	0.780
–	–	*S. aureus* Newman	0.780

**Fig. 1.**
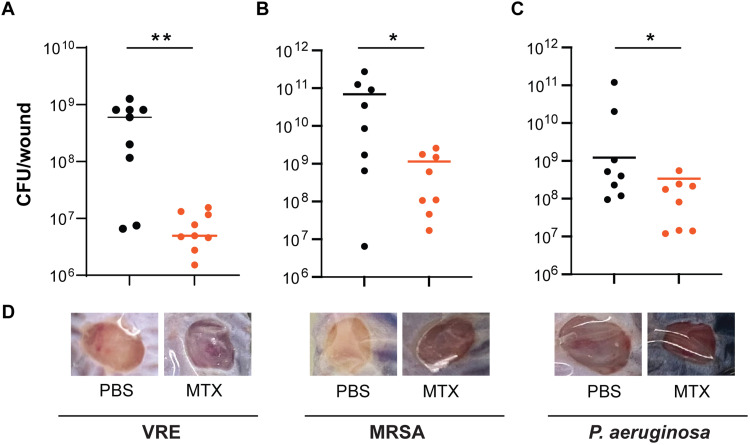
MTX exhibits potent antibiotic activity in vivo. (**A** to **C**) Comparison of VRE (A), MRSA (B), and *P. aeruginosa* (C) CFU per infected wound treated with either PBS (black) or MTX (orange). Each symbol represents one mouse with the median indicated by the horizontal line. Data were from two independent experiments with four to five mice per experiment. Statistical analysis was performed using the nonparametric Mann-Whitney test to compare ranks; *P ≤ 0.05 and **P ≤ 0.01. (**D**) Representative images of VRE-, MRSA-, and *P. aeruginosa*–infected wounds treated with PBS or MTX.

We tested the potency of MTX in inhibiting bacterial growth in vivo using a mouse wound infection model (*15*). Wounds were infected with 10^7^ colony-forming units (CFU) of VRE, followed with addition of 10 μl of MTX (0.515 μg/ml) or phosphate-buffered saline (PBS). Twenty-four hours post-infection (hpi), the median CFU per wound was 6 × 10^8^ for PBS controls, whereas the median CFU was 4.9 × 10^6^ for MTX-treated wounds, a ~120-fold lower CFU than the PBS control (Fig. 1A). Similarly, MTX treatment reduced the median CFU of MRSA USA300 and *P. aeruginosa* PAO1 by ~60- and ~3.5-fold (Fig. 1, B and C), respectively. Moreover, infected wounds were presented with a purulent exudate, which was visibly reduced with MTX treatment, most prominently for VRE and *S. aureus* infection (Fig. 1D). These results show that MTX exhibits potent antibiotic activity both in vitro and in vivo, especially against Gram-positive bacterial species, including those resistant to vancomycin.

### MTX synergizes with vancomycin to inhibit VRE in vitro and in vivo

**Fig. 2.**
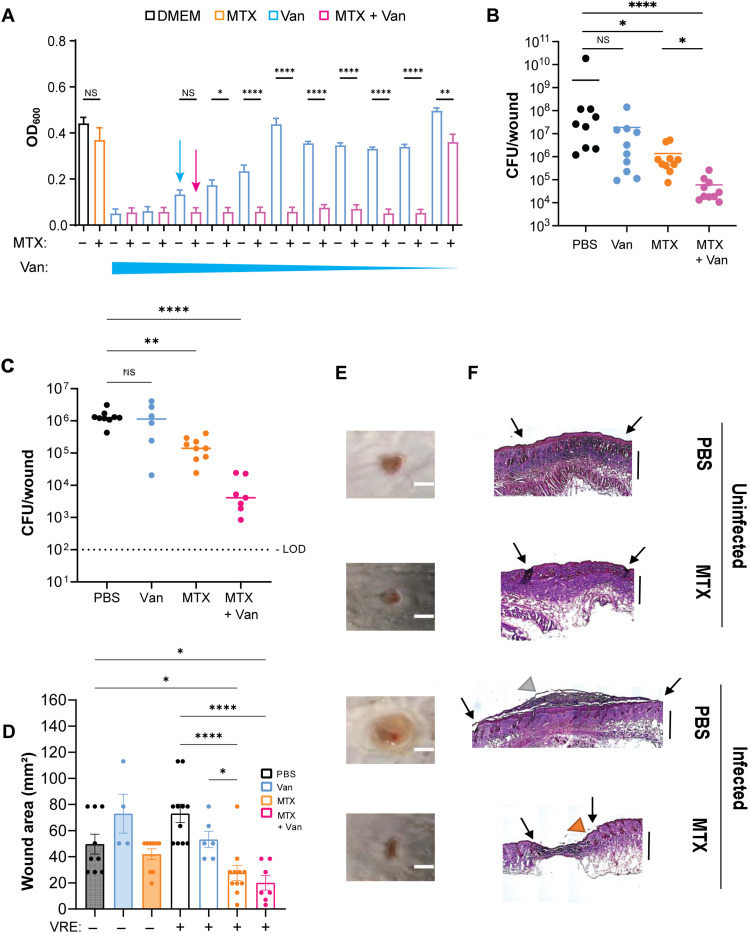
MTX and vancomycin synergize to inhibit VRE in vitro and in vivo. (**A**) Comparison of VRE growth in DMEM, in the presence of MTX (0.515 μg/ml), in the presence of decreasing concentrations (75 to 0.0625 μg/ml) of vancomycin, and with a combination of MTX and vancomycin. Arrows represent the breaking point where vancomycin concentration alone (18 μg/ml) starts to differ from vancomycin concentration in the presence of MTX. Data (mean ± SEM) were derived from three independent experiments. (**B**) Comparison of VRE CFU per wound after treatments. (**C**) Comparison of VRE CFU per wound after multiple treatments. Vancomycin treatment was performed only in the first day. The limit of detection (LOD) is shown. Data in (B) and (C) were from two independent experiments with three to four mice per experiment. Each symbol represents one mouse, with the median indicated by the horizontal line. Statistical analysis was performed using Kruskal-Wallis test with uncorrected Dunn’s posttest. (**D**) Wound area measured at 4 days post-infection (dpi) after five treatments. Vancomycin treatment was performed only in the first day, alone or in combination with MTX. Statistical analysis was performed in (A) and (D) using ordinary one-way ANOVA, followed by Tukey’s multiple comparison test. For all analyses, NS denotes not significant; *P ≤ 0.05, **P ≤ 0.01, and ****P ≤ 0.0001. (**E**) Representative images of wounds at the end of the multiple-treatment experiment. Scale bars, 5 mm. (**F**) Wounds were harvested at 4 dpi and subjected to H&E staining. The wound edges (black arrows), thickened epidermis (gray arrowhead), and normal margin (orange arrowhead) are shown. Scale bars, 400 μm.

We investigated whether MTX synergizes with vancomycin in inhibiting the growth of VRE using a modified MIC assay in which the concentration of MTX was kept constant at sub-MIC (0.515 μg/ml) (Fig. 2A and fig. S2A), while the concentrations of vancomycin were increased by twofold from 0.0625 to 75 μg/ml. As expected, VRE started to grow just below its breakpoint of 18 μg/ml of vancomycin and grew without any inhibition at 1.5 μg/ml of vancomycin (Fig. 2A). However, the growth of VRE was potently inhibited at 0.125 μg/ml of vancomycin in the presence of sub-MIC MTX (Fig. 2A), indicating a 140-fold reduction of vancomycin MIC in the presence of sub-MIC MTX (table S2). Combinatorial MTX and vancomycin treatment was initially bacteriostatic but became bactericidal by 24 hours (fig. S2B). Similarly, we tested combination of MTX (0.515 μg/ml) with other antibiotics against VRE. In the presence of MTX, the MIC for ceftriaxone, daptomycin, ciprofloxacin, chloramphenicol, and penicillin G was reduced by eight-, eight-, four-, four-, and twofold, respectively (table S2), suggesting a general enhancement against VRE.

We further tested the synergy between MTX and vancomycin in the wound infection model. Mice were first intraperitoneally injected with 100 mg/kg (human equivalent dose of ~8 mg/kg) of vancomycin, which was lower than the minimum equivalent recommended dose (>10 mg/kg) to treat Gram-positive bacterial infections in humans (*26*). Then, mice were subjected to wound infection with VRE and a single MTX treatment as described in Fig. 1A. As shown in Fig. 2B, vancomycin alone reduced the median CFU by 11-fold and MTX alone reduced the median CFU by 42-fold, whereas vancomycin and MTX combination reduced the median CFU by 1000-fold. Thus, MTX and vancomycin synergize to inhibit VRE both in vitro and in vivo.

We also evaluated the effect of multiple treatment of MTX in vivo. Following a single intraperitoneal injection of vancomycin, mice were subjected to wound infection with VRE and the first dose of MTX treatment, as described above. Then, a daily MTX treatment at the same dose was applied onto the infected wound for the next 4 days. This multidose MTX treatment regimen reduced the bacterial burden in the wound by 33-fold compared to multidose MTX treatment in the absence of vancomycin (Fig. 2C). Multidose MTX treatment without vancomycin also reduced the bacterial burden by nearly 10-fold compared to a single-dose MTX treatment (Fig. 2, B and C). When in combination with vancomycin, multiple MTX treatments resulted in 14-fold fewer CFU compared to wounds treated a single time with both MTX and vancomycin (Fig. 2, B and C). Moreover, multiple MTX treatment accelerated wound closure in comparison to infected PBS controls (Fig. 2, D and E). Previous observations that *E. faecalis* infection delays wound closure and leads to thickened epidermis (*15*) were confirmed by hematoxylin and eosin (H&E) staining of wound sections treated with PBS (Fig. 2F). MTX-treated wounds, on the other hand, despite also presenting thickened epidermis at the center of the wound, probably because of infection, had clear skin margins and layers, indicating proper healing (Fig. 2F).

### MTX kills VRE by inducing reactive oxygen species and DNA damage

**Fig. 3.**
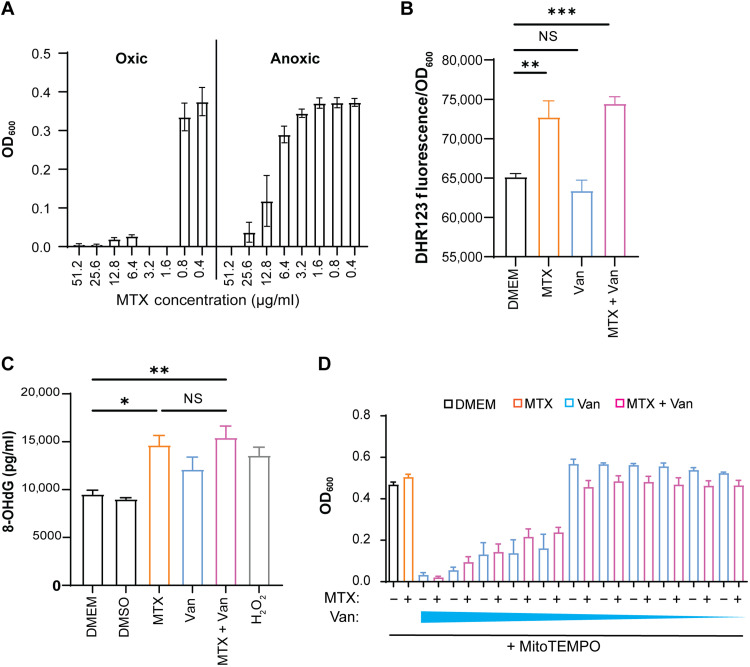
MTX induces production of ROS and DNA damage in bacterial cells. (**A**) Comparison of VRE growth (OD_600_) in oxic and anoxic conditions in the presence of decreasing concentrations of MTX. Data (mean ± SEM) are summary of three independent experiments. (**B**) Comparison of intracellular ROS levels, as measured by DHR123 fluorescence, in VRE cultures treated with MTX (0.515 μg/ml), vancomycin (4 μg/ml), or both. (**C**) Comparison of 8-OHdG levels, as measured by ELISA, in VRE cultures treated with MTX (0.515 μg/ml), vancomycin (4 μg/ml), or both. H_2_O_2_ (0.1 mM) was added into the VRE culture as a positive control. Data (mean ± SEM) in (C) and (D) are summary from three independent experiments each. Statistical analysis was performed using ordinary one-way ANOVA, followed by Tukey’s multiple comparison test; NS, *P* > 0.05; *P ≤ 0.05, **P ≤ 0.01, and ***P ≤ 0.001. (**D**) Comparison of VRE growth in DMEM (black), in the presence of MTX (0.515 μg/ml) (orange), in the presence of decreasing concentrations (75 to 0.0625 μg/ml) of vancomycin (blue), and with a combination of MTX and vancomycin (pink). MitoTEMPO was added into all cultures. Data (mean ± SEM) were derived from three independent experiments for each sample per experiment.

MTX has been shown to stimulate the formation of reactive oxygen species (ROS) in hepatocytes (*27*). Because ROS can kill bacteria directly (*28*), we examined the role of ROS in MTX-mediated inhibition of bacterial growth by measuring the MIC of MTX under oxic and anoxic conditions. Under oxic conditions, VRE growth was completely inhibited by MTX at 1.6 μg/ml, whereas under anoxic conditions, 51.2 μg/ml of MTX was required to completely inhibit VRE growth (Fig. 3A), a 32-fold decrease in MTX MIC in the presence of oxygen. Quantification of intracellular ROS production using dihydrorhodamine 123 (DHR123) revealed that the sub-MIC of MTX (0.515 μg/ml), but not vancomycin (4 μg/ml), induced significant ROS production (Fig. 3B). Consistently, MTX also induced elevation of 8-hydroxy-2′-deoxyguanosine (8-OHdG), a by-product of ROS-induced DNA damage, to a level similar to that produced by 0.1 mM H_2_O_2_ (Fig. 3C). Addition of vancomycin to MTX-treated cultures did not further increase the levels of ROS and 8-OHdG, suggesting that vancomycin may increase the speed of MTX uptake and ROS induction, rather than the overall amount of ROS produced. Furthermore, when the ROS scavenger MitoTEMPO was added to VRE cultures, VRE growth in the presence of vancomycin plus MTX was similar to that in the presence of vancomycin alone (Fig. 3D), indicating that the effect of MTX in synergizing with vancomycin was completely abolished by ROS scavenger. In addition, MTX alone or in combination with vancomycin significantly up-regulated the transcript level of the oxidative stress response gene *sodA*, but not significantly *groES*, *lexA*, or *recA* in VRE (fig. S3). These results suggest that induction of ROS and DNA damage is a key mechanism by which MTX exhibits antibiotic activity and synergizes with vancomycin.

### Vancomycin-treated VRE displays increased permeability to MTX

**Fig. 4.**
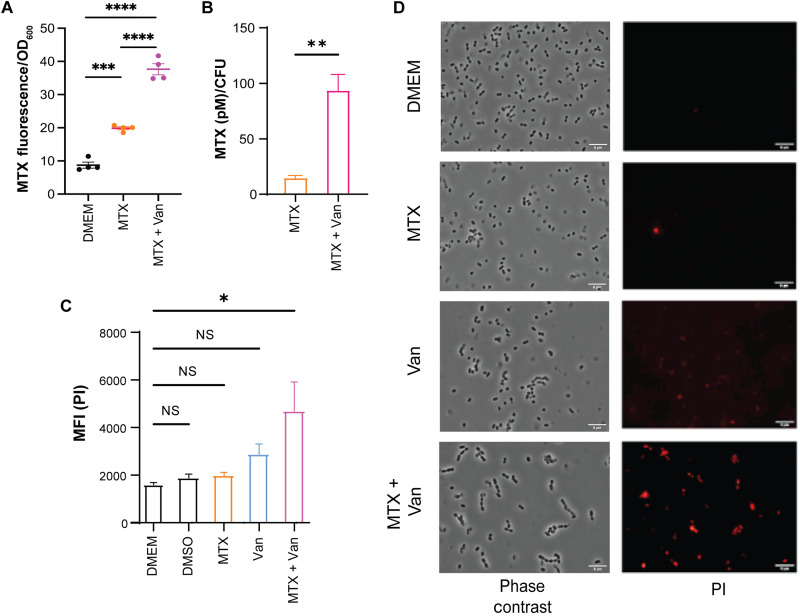
Vancomycin-treated VRE bacterial cells have increased permeability to MTX. (**A**) MTX uptake by VRE after 6 hours of treatment with MTX (0.515 μg/ml) alone and in combination with vancomycin (4 μg/ml). Each dot represents one independent experiment. (**B**) Mass spectrometry quantification of intracellular MTX of VRE cultures treated with MTX (0.515 μg/ml) and in combination with vancomycin (4 μg/ml) for 1 hour. Data (mean ± SEM) are summary of five independent replicates. Statistical analysis was performed using unpaired *t* test with Welch’s corrections; **P ≤ 0.01. (**C**) Comparison of PI uptake by VRE after 6 hours of treatment with MTX (0.515 μg/ml) alone, vancomycin alone (4 μg/ml), or in combination. (**D**) Epifluorescence microscopy images of VRE stained with PI after 6 hours of treatment with MTX, vancomycin, or both. Scale bars, 5 μm. (A and C) Data (mean ± SEM) are summary of at least three independent experiments. Statistical analysis was performed using ordinary one-way ANOVA, followed by Tukey’s multiple comparison test; NS, *P* > 0.05; *P ≤ 0.05, **P ≤ 0.01, ***P ≤ 0.001, and ****P ≤ 0.0001.

We noticed that MTX lowered the sensitivity of VRE strains to vancomycin but did not further sensitize vancomycin-susceptible *E. faecalis* or other vancomycin-susceptible bacterial species (Table 2), suggesting that the bacterial vancomycin resistance mechanism itself might play a role in the observed synergistic effect between sub-MIC doses of MTX and vancomycin. Vancomycin interferes with bacterial cell wall synthesis and therefore increases cell wall permeability (*29*). We tested whether vancomycin increases the uptake of MTX by measuring MTX’s fluorescence emission at 685 nm (*30*). As shown in Fig. 4A, the fluorescence intensity of VRE doubled in the presence of MTX as compared to medium alone. The fluorescence intensity quadrupled when VRE were treated with both MTX and vancomycin. Consistently, mass spectrometry quantification of intracellular MTX showed that MTX levels were about six times higher in VRE cultures in the presence of both MTX and vancomycin than MTX alone (Fig. 4B). Moreover, more propidium iodide (PI) was taken into VRE at 6 hours following treatment with both MTX and vancomycin, as measured by flow cytometry (Fig. 4C) and fluorescence microscopy (Fig. 4D) compared to MTX treatment alone. VRE cultures treated with both MTX and vancomycin and grown under anoxic conditions also displayed elevated uptake of MTX, suggesting that the increased MTX MIC observed in anoxic conditions is not due to changes in bacterial cell wall or metabolism that prevent MTX uptake but is more likely due to MTX’s reduced ability to induce ROS under this condition (fig. S4). These results show that vancomycin increases the uptake of MTX probably due to its interference with bacterial cell wall synthesis, i.e., the resistance mechanism itself in VRE strains.

**Table 2.** Vancomycin-resistant strains have lower vancomycin MIC in the presence of a sub-inhibitory dose of MTX.

Strain	Vancomycin MIC* (μg/ml)	Vancomycin MIC + MTX (0.515 μg/ml)
*E. faecalis* V583 (VRE)	18.00	0.125
*E. faecium* AUS0004 (VRE)	4.68	<0.14
*E. faecium* E745 (VRE)	75.00	4.68
*E. faecium* E8252 (VRE)	37.5	4.68
*E. faecalis* OG1RF	4.00	4.00
*S. aureus* USA300	2.00	2.00
*E. faecalis* 12030	3.75	3.75
*E. faecalis* TTSHW-EF43	3.75	1.88
*E. faecalis* ATCC 29121	3.75	3.75
*S. aureus* 8325-4	1.88	1.88
*S. aureus* MN8	7.50	7.50
*S. aureus* 15981	3.75	3.75
*S. aureus* ISP479	7.50	7.50
*S. aureus* Newman	3.75	3.75
*E. coli* 958	512	512
*P. aeruginosa* PAO1	800	800
*S. Typhimurium* 14028S	800	800

*MIC was established by measuring OD_600_ in the presence of decreasing concentrations of vancomycin with or without MTX.

To further test this possibility, we expressed the *van* operon, without the genes encoding the vancomycin-sensing VanRS two-component system, from *E. faecalis* V583 into OG1RF under the control of a nisin-inducible promoter. As expected, the resulting OG1RF harboring the *van* operon was more resistant to vancomycin compared to the parental OG1RF. Without nisin induction, OG1RF harboring the *van* operon was as sensitive to vancomycin as the parental OG1RF (Table 3). However, with nisin induction, OG1RF harboring the *van* operon was eightfold more sensitive to vancomycin compared to the uninduced condition in the presence of MTX (Table 3), suggesting that the cellular changes that occur when vancomycin is present and that lead to vancomycin resistance enable increased permeability to MTX. Together, these data suggest that the synergy between MTX and vancomycin is due to vancomycin-induced uptake of MTX, which, in turn, kills bacteria by inducing ROS and DNA damage.

**Table 3.** Vancomycin or MTX MIC of *E. faecalis* OG1RF harboring different plasmid constructs.

Strain	Vancomycin MIC* (μg/ml)		Vancomycin MIC + MTX (0.515 μg/ml)	
	−	+	−	+
*E. faecalis* OG1RF WT	4	4	4	4
*E. faecalis* OG1RF *+* pMSP3535*::*VRE*van*operon	4–8	16	4	0.5
**Strain**		**MTX MIC^†^ (μg/ml)**		
*E. faecalis* OG1RF WT		0.604		
*E. faecalis* OG1RF + VRE *dead/deah*		1.208		
*E. faecalis* OG1RF *+* VRE MTX^R^ *dead/deah*		4.832		
*E. faecalis* OG1RF + VRE EF_RS05155		0.604		
*E. faecalis* OG1RF *+* VRE MTX^R^ EF_RS05155		0.604		
*E. faecalis* OG1RF + VRE EF_RS07835		0.604		
*E. faecalis* OG1RF *+* VRE MTX^R^ EF_RS07835		0.604		
*E. faecalis* OG1RF + VRE EF_RS09020		0.604		
*E. faecalis* OG1RF *+* VRE MTX^R^ EF_RS09020		0.604		

*Vancomycin MIC of *E. faecalis* OG1RF expressing the *van* operon under an inducible promoter in the absence (−) or presence (+) of nisin. MIC was established by measuring OD_600_ in the presence of decreasing concentrations of vancomycin with or without MTX and with (+) or without (−) induction with nisin (50 ng/ml). Erythromycin was kept at 25 μg/ml for all tested conditions with strain *E. faecalis* OG1RF *+* pMSP3535*::*VRE*van*operon because pMSP3535 confers resistance to this antibiotic. Nisin alone did not influence MTX MIC of OG1RF strain.

† MTX MIC of *E. faecalis* OG1RF and *E. faecalis* OG1RF constitutively expressing either the wild-type or the mutant copy of the mutated genes of strain VRE MTX^R^. MIC was established by OD_600_ to measure bacterial growth in the presence of decreasing concentrations of MTX (19.31 to 0.07 μg/ml).

### Mutation in a DEAD/DEAH box helicase confers resistance to MTX

**Fig. 5.**
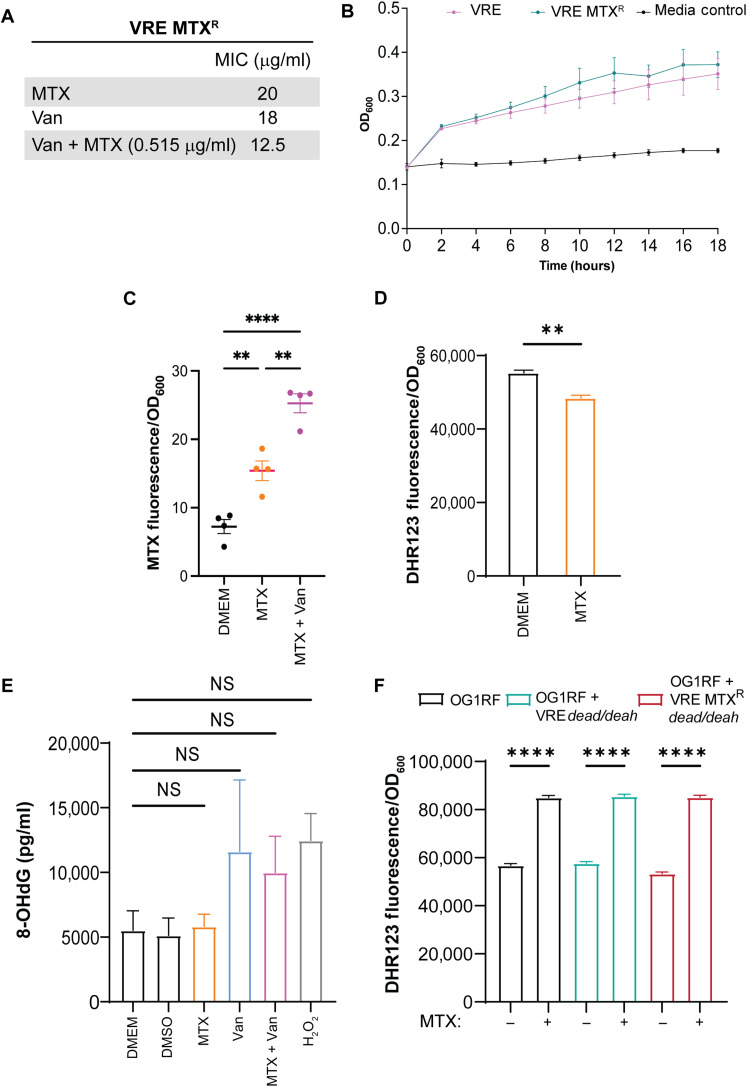
VRE MTX^R^ exhibits increased MTX uptake but not increased ROS production and DNA damage. (**A**) Comparison of MIC for MTX alone, vancomycin alone, and vancomycin in the presence of MTX (0.515 mg/ml) between the parental VRE and VRE MTX^R^. Each dot represents one independent experiment. (**B**) Growth curve of VRE and VRE MTX^R^ in DMEM. (**C**) MTX uptake by VRE MTX^R^ after 6 hours of treatment with MTX (0.515 μg/ml) alone and in combination with vancomycin (4 μg/ml). (**D**) Analysis of ROS levels in VRE MTX^R^ treated with MTX (0.515 μg/ml). (**E**) ELISA measurements of 8-OHdG levels in VRE MTX^R^ treated with MTX (0.515 μg/ml) and vancomycin (4 μg/ml), separately and in combination, and the positive control H_2_O_2_ (0.1 mM). (**F**) Analysis of ROS levels in *E. faecalis* OG1RF and *E. faecalis* OG1RF constitutively expressing either a wild-type (VRE *dead/deah*) or the mutant DEAD/DEAH helicase (VRE MTX^R^ *dead/deah*) treated with MTX (0.515 μg/ml). (C to F) Data (mean ± SEM) are summary of at least three independent experiments. Statistical analysis was performed using ordinary one-way ANOVA, followed by Tukey’s multiple comparison test; NS, *P* > 0.05; **P ≤ 0.01, and ****P ≤ 0.0001.

To further investigate the mechanism by which MTX inhibits bacterial growth, we performed in vitro evolution to select for spontaneous mutants that were resistant to MTX. We serially passaged VRE in medium with increasing concentrations of MTX from a sub-MIC concentration of 0.515 to 2.84 μg/ml. Among 16 colonies obtained, one exhibited the highest MTX resistance at MIC of 20 μg/ml (Fig. 5A and table S3) or a 12.5-fold increase over the parental VRE. This mutant, henceforth named as VRE MTX^R^, was as sensitive to vancomycin alone as the parental strain (MIC = 18 mg/ml) but was over 100-fold more resistant to vancomycin in the presence of MTX (MIC = 12.5 μg/ml). Nevertheless, VRE MTX^R^ had similar growth kinetics as the parental VRE (Fig. 5B and fig. S5). Whole-genome sequencing revealed the presence of four mutations in the genome of VRE MTX^R^ (table S3). Among them, a point mutation (G → A) occurred in a gene predicted to encode a DEAD/DEAH box helicase, resulting in a substitution of glycine (G) by arginine (R) at the amino acid position 389.

RNA helicases of the DEAD/DEAH box family have been linked to oxidative stress resistance in bacteria (*31*). Compared to the parental VRE, VRE MTX^R^ exhibited a similar increase in MTX uptake in the presence of vancomycin (Fig. 5C). However, in contrast to the parental VRE, MTX treatment did not increase the intracellular ROS or 8-OHdG in VRE MTX^R^ (Fig. 5, D and E). VRE MTX^R^ displayed a lower baseline DNA damage or DNA damage in response to H_2_O_2_ (Fig. 5E), as compared to the parental VRE (Fig. 3D). These results show that MTX does not induce ROS and DNA damage in the mutant VRE, likely explaining its increased resistance to MTX.

To determine whether the DEAD/DEAH box helicase could confer OG1RF resistance to MTX, we found that the vancomycin-sensitive *E. faecalis* strain OG1RF does not have a homolog of the DEAD/DEAH box helicase gene based on BLASTp and BLASTn analysis. We constitutively expressed either the wild-type (WT) or the mutant DEAD/DEAH box helicase gene in OG1RF. Despite an increase in the intracellular ROS upon MTX exposure in all strains (Fig. 5F), *E. faecalis* OG1RF harboring the WT gene (VRE *dead/deah*) and the mutated copy of DEAD/DEAH box helicase gene (VRE MTX^R^ *dead/deah*) was two- and eightfold more resistant to MTX than the parental OG1RF strain, respectively (Table 3). In contrast, *E. faecalis* OG1RF harboring the WT and mutated copy of the other three genes found to be mutated in VRE MTX^R^ was not more resistant to MTX (Table 3). Thus, the DEAD/DEAH box helicase plays a critical role in protecting the VRE MTX^R^ from the effects of MTX by reducing the damage caused by ROS.

### MTX enhances macrophages to eliminate bacterial infection in vivo

**Fig. 6.**
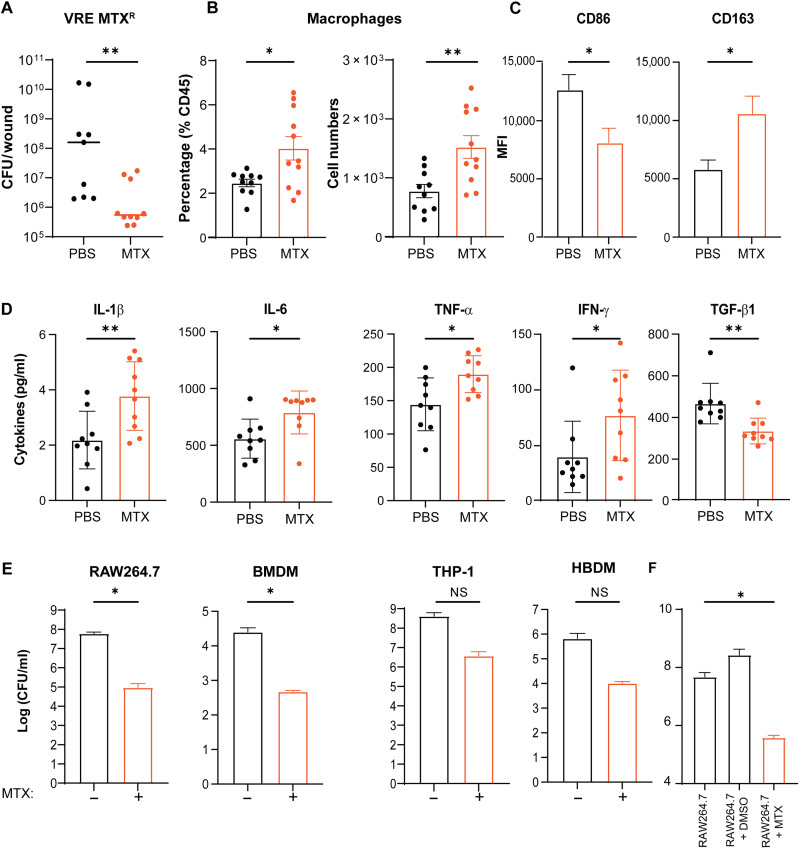
MTX treatment promotes macrophage recruitment and reprogramming to a proinflammatory phenotype. VRE MTX^R^–infected wounds were treated for 24 hours with either PBS or a single dose of MTX [10 μl of MTX (0.515 μg/ml) per wound]. (**A**) Comparison of VRE MTX^R^ CFU in wound lysates treated with either PBS or MTX for 24 hpi. Data (mean ± median) are a summary of two independent experiments with four to five mice per group. (**B**) Percentage and absolute numbers of macrophages recovered from infected wounds treated with PBS or MTX. Data (mean ± SEM) are summary of two independent experiments. Each dot represents one mouse. (**C**) Comparison of mean fluorescence intensity (MFI) of CD86 and CD163 staining gating on CD45^+^ CD11b^+^ F4/80^+^ macrophages from infected wounds treated with PBS or MTX. Data (mean ± SEM) are from five mice per group. (**D**) Levels of cytokines IL-1β, IL-6, TNF-α, IFN-γ, and TGF-β from the lysates of infected wound treated with PBS or MTX. Data (mean ± SEM) are summary of two independent experiments. Each dot represents one mouse. (**E**) Comparison of VRE CFU counts in RAW264.7, BMDM, THP-1, and HBDM (human blood-derived macrophage) in the presence or absence of MTX. Data (mean ± SEM) are summary of three independent experiments. (**F**) VRE CFU performed with MTX-pretreated RAW264.7 macrophage cells. Data (mean ± SEM) are summary of at least three independent experiments. Statistical analysis was performed using the nonparametric Mann-Whitney test to compare ranks (A), unpaired *t* test with Welch’s corrections (B to E), or ordinary one-way ANOVA followed by Tukey’s multiple comparison test (F). NS, *P* > 0.05; *P ≤ 0.05 and **P ≤ 0.01.

We have previously shown that MTX can reprogram macrophages toward a proinflammatory phenotype (*25*), which could lead to more effective bacterial killing. Therefore, we investigated whether MTX immunomodulatory activity contributes to reduced bacterial CFU in infected wounds in vivo. To distinguish between antibiotic and immunomodulatory effects of MTX, we infected wounds with VRE MTX^R^ together with MTX or PBS. MTX treatment resulted in ~290-fold fewer CFU compared to the PBS control (Fig. 6A). Because the reductions in CFU were comparable (290-fold versus 120-fold) when VRE- and VRE MTX^R^–infected wounds were treated with the same amount of MTX, and because VRE MTX^R^ is resistant to MTX, the reduction of VRE MTX^R^ CFU is likely to be largely due to MTX immunomodulatory rather than antibiotic activity. Consistently, concomitant treatment of VRE MTX^R^–infected wounds with MTX and vancomycin led to about eightfold reduction of CFU as compared to MTX or vancomycin treatment alone (fig. S6), indicating that the synergy in vivo is primarily due to the immunomodulatory effects of MTX, independent of MTX resistance and MTX-mediated killing.

To provide further support, we quantified macrophages and neutrophils in the wounds 24 hours after VRE infection and MTX treatment. VRE-infected wounds treated with MTX contained twice the number of macrophages compared to that from infected wounds treated with PBS (Fig. 6B and fig. S7A). Similarly, a significant increase in the percentage of neutrophils was also observed in VRE-infected wounds treated with MTX (fig. S7B). Flow cytometry analysis of the infection-related markers CD163, CD86, CD206, and major histocompatibility complex II (MHCII) (fig. S7, C and D) (*32*) showed that macrophages expressed a higher level of CD163, the high-affinity scavenger receptor, but expressed a lower level of CD86 in VRE-infected wounds treated with MTX as compared to PBS treatment (Fig. 6C). Increased macrophage recruitment to the MTX-treated infected wounds correlated with significant increases in the levels of the proinflammatory cytokines interleukin-1β (IL-1β), IL-6, tumor necrosis factor–α (TNF-α), and interferon-γ (IFN-γ) in MTX-treated wounds and a lower level of the anti-inflammatory cytokine transforming growth factor–β1 (TGF-β1) (Fig. 6D). Consistently, MTX treatment of infected RAW264.7 macrophages resulted in significantly higher nuclear factor κB (NF-κB)–driven transcription than the PBS-treated infected cells (fig. S7E).

We also examined whether MTX promotes macrophage killing of intracellular bacteria. RAW264.7 macrophages and primary murine bone marrow–derived macrophages (BMDMs) were infected with VRE for 3 hours followed by 15 hours of combined treatment with MTX and gentamicin plus penicillin, which eliminate any residual extracellular bacteria. MTX treatment resulted in ~3-log fewer intracellular CFU compared to untreated infected cells at 18 hpi (Fig. 6E). Similarly, MTX promoted intracellular killing of VRE in the human monocyte-like cell line (THP-1) and primary human monocyte-derived macrophages (HMDMs) (Fig. 6E), although the difference was not statistically significant. Moreover, MTX also enhanced macrophage killing of both Gram-positive and Gram-negative bacteria, including *E. faecium*, *S. aureus*, *P. aeruginosa*, and multidrug-resistant *Escherichia coli* EC958 (fig. S8), and neutrophil killing of the VRE strain in vitro (fig. S9).

To exclude a direct antibiotic effect of MTX on intracellular bacterial killing, we pretreated RAW264.7 macrophages with MTX overnight, washed cells to remove residual MTX in the culture, and then infected with VRE. The pretreatment reduced intracellular CFU by ~2-log by 18 hpi (Fig. 6F). The same infection and MTX pretreatment did not lead to host cell death (fig. S7F) or host cell membrane permeability (fig. S7G). Similarly, wounds that were pretreated with a single dose of MTX had 20-fold fewer CFU compared to the PBS control (fig. S10). Together, these results show that MTX promotes macrophage recruitment to the site of infection in vivo and reprograms macrophages to proinflammatory phenotypes to eliminate bacteria more efficiently.

### MTX enhances bactericidal activity of macrophages by stimulating lysosomal enzyme expression and activity

To determine how MTX enhances macrophage bacterial killing, we investigated whether MTX stimulates macrophage phagocytosis of bacteria. RAW264.7 macrophage cells were treated with MTX for 16 hours and then incubated with fluorescent fixed VRE. After 3 hours of incubation, the fluorescence of extracellular bacteria was quenched with trypan blue, and phagocytosis of fluorescent bacteria was measured by flow cytometry and visualized by confocal microscopy. Fluorescent bacteria were visible inside RAW264.7 cells, but there was no significant difference in phagocytosis by RAW264.7 with or without MTX treatment (fig. S11, A to C). We also tested whether MTX stimulates macrophages to produce ROS. In the absence of infection, MTX did not induce ROS production by RAW264.7 (fig. S11D). Following VRE infection, RAW264.7 cells produced ROS regardless of MTX addition. Consistently, addition of the superoxide scavenger MitoTEMPO did not reduce bactericidal activity of macrophages (fig. S11E). These results suggest that neither increased phagocytosis nor ROS production explains why MTX enhances macrophage bactericidal activity.

**Fig. 7.**
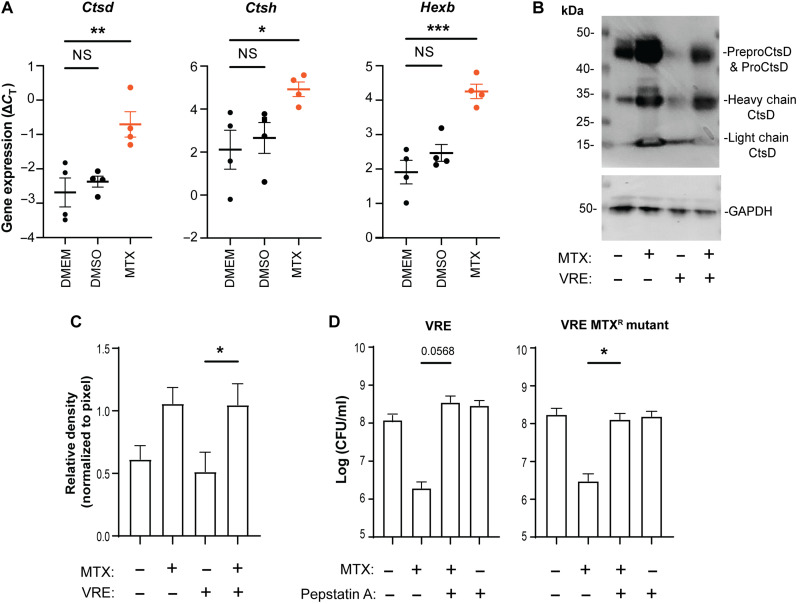
MTX enhances macrophage antimicrobial activity by stimulating lysosomal enzyme expression and activity. (**A**) qRT-PCR analysis of *Ctsd*, *Ctsh*, and *Hexb* transcript levels (Δ*C*_T_) in RAW264.7 cells with or without DMSO or MTX treatment overnight. Each dot represents one biological replicate. (**B** and **C**) Western blotting analysis of whole-cell lysates with anti–cathepsin D antibody. RAW264.7 cells with (+) and without (−) VRE infection were treated with MTX (+) or left untreated (−). Whole-cell lysates were separated by SDS–polyacrylamide gel electrophoresis, transferred to membrane, and probed with anti–cathepsin D antibody or anti-GAPDH (control) (B). Relative band density of the CtsD heavy chain normalized to that of GAPDH (C). (**D**) RAW264.7 cells were infected with either VRE or VRE MTX^R^ in the presence of MTX (0.515 μg/ml), pepstatin A (10 μg/ml), or both. Intracellular bacterial CFU was quantified. Data (mean ± SEM) are a summary of at least three independent experiments. Statistical analysis was performed using ordinary one-way ANOVA, followed by Tukey’s multiple comparison test; NS, *P* > 0.05; *P ≤ 0.05, **P ≤ 0.01, and ***P ≤ 0.001.

We investigated whether MTX enhances macrophage killing of bacteria by stimulating lysosomal activity. We quantified the transcript levels of 16 lysosomal pathway genes by quantitative reverse transcription polymerase chain reaction (qRT-PCR) in uninfected RAW264.7 macrophages following MTX treatment for 24 hours (fig. S12). Transcripts for lysosomal proteases cathepsins D (*Ctsd*) and H (*Ctsh*) and the enzyme β-hexosaminidase (β subunit) (*Hexb*), which cleaves glycosides, were up-regulated (Fig. 7A and fig. S12). We further verified the induction of CtsD protein in MTX-treated RAW264.7 cells in both the presence and absence of bacterial infection by Western blotting (Fig. 7, B and C). Notably, fully processed CtsD heavy and light chains were abundant (Fig. 7C), indicating activation of CtsD enzymatic activity in MTX-treated macrophages. To directly test the role of CtsD and other lysosomal proteases in bactericidal activity, we quantified the intracellular bacteria in the presence of lysosomal protease inhibitor pepstatin A. While pepstatin A alone did not inhibit macrophage killing of intracellular VRE or VRE MTX^R^, it completely abolished MTX-stimulated macrophage killing of intracellular bacteria (Fig. 7D). These results show that MTX stimulates macrophages to kill bacteria by up-regulating lysosomal enzyme expression and activity.

## DISCUSSION

Ever-increasing antibiotic resistance requires new approaches that leverage both antibiotics and the immune system in combating bacterial infection. In this study, we investigated MTX, a chemotherapeutic initially approved for treating acute myeloid leukemia, for its antibiotic activity and its ability to recruit and activate macrophages for bacterial clearance. We show that (i) MTX has potent antibiotic activity against Gram-positive bacteria, (ii) MTX and vancomycin synergize to overcome vancomycin resistance, and (iii) sub-MIC levels of MTX are sufficient to recruit and activate macrophages to clear bacteria in a mouse model of wound infection. We determined the molecular mechanisms that underlie MTX antibiotic activity, synergy with vancomycin, and activation of macrophage bactericidal activity.

MTX was previously reported to exhibit antibiotic activity in MIC tests performed in nutrient-rich medium against *Streptococcus pneumoniae* and *S. aureus* (*33*, *34*). Here, we tested MTX antibiotic activity against a panel of Gram-positive and Gram-negative bacterial species and strains and found MTX to be 10- to 20-fold more potent against Gram-positive bacteria with MIC of ~1 μg/ml. We also show that sub-MIC MTX reduced the growth of a vancomycin-resistant strain of *E. faecalis* (VRE) by ~100-fold in a murine wound infection model. To our knowledge, this is the first time MTX has been used topically to treat bacterial infections. Topical use at a low dose may limit the potential side effects of MTX administered systemically.

As a chemotherapeutic, MTX is known to induce DNA damage as well as free radical formation and lipid peroxidation in eukaryotic cells (*27*). As an antibiotic, it was previously proposed that its main mode of action was inhibition of bacterial DNA gyrase (*34*, *35*). Here, we show that MTX kills bacteria primarily by induction of ROS and DNA damage with three lines of evidence. First, the antibiotic activity of MTX is over 30-fold stronger in the presence of oxygen than in the absence of oxygen. Second, a sub-MIC dose of MTX is sufficient to cause significant ROS elevation and DNA damage in the bacterial cells. Third, addition of superoxide scavenger MitoTEMPO to the culture completely abolished MTX’s synergistic antibiotic activity with vancomycin. This mechanism of action is supported by the generation of an MTX-resistant mutant VRE, carrying a mutation in the gene encoding a predicted DEAD/DEAH box helicase. Enzymes of the DEAD/DEAH box family are RNA helicases implicated in many processes (*33*, *41*), including oxidative stress resistance in some bacterial species (*31*). Compared to the parental VRE, VRE MTX^R^ had similar uptake of MTX, but produced lower levels of ROS, and had significantly reduced DNA damage. Furthermore, heterologous expression of the VRE WT and mutant DEAD/DEAH box helicase genes in *E. faecalis* OG1RF, which lacks this gene, was sufficient to confer MTX resistance by two- and eightfold, respectively. These results suggest that the induction of ROS and DNA damage is a key mechanism by which MTX exhibits antibiotic activity.

Clinically, combinatorial therapy using antibiotics with different mechanisms of action and different targets is used to prevent antibiotic resistance (*36*). In this study, we found that MTX and vancomycin are a highly effective combination against vancomycin-resistant strains both in in vitro culture and in a murine model of wound infection. The observed synergy was limited to vancomycin-resistant strains, not vancomycin-sensitive strains, because the vancomycin-resistant cells display increased permeability to MTX, thereby enabling elevated ROS production and DNA damage and therefore synergy. It is probable that, in VRE strains, vancomycin induces the resistance mechanism, leading to cell wall remodeling, which, in turn, facilitates MTX uptake (fig. S13). It remains to be determined whether vancomycin bound to unmodified PG subunits, before VanRS-induced peptide alteration from d-Ala-d-Ala to d-Ala-d-Lac, also contributes to increased permeability, cell wall remodeling, and, ultimately, the observed synergy phenotype. This finding may have broader implications for the treatment of vancomycin-resistant bacterial infections and suggests the possibility of combination therapies where vancomycin may act as a permeability enhancer in strains otherwise resistant to this antibiotic.

In addition to having direct antibiotic activity, MTX also stimulates macrophages to more effectively clear bacteria both in vitro and in vivo. Because augmented macrophage killing activity occurred with sub-MIC concentrations of MTX, we conclude that MTX acts primarily by activating macrophages, rather than its antibiotic activity. Consistent with this interpretation, MTX-stimulated macrophage killing of bacteria is as effective for Gram-positive as for Gram-negative bacteria both in vitro and in vivo. Pretreatment of RAW264.7 macrophage-like cells with MTX enhanced VRE killing, indicating reprogramming of macrophages for enhanced bactericidal activity. Furthermore, MTX-stimulated macrophages clear VRE MTX^R^ nearly as efficiently as the parental VRE strain in the wound infection, further supporting that reprogramming macrophages by MTX promotes bacterial clearance.

ROS and lysosomal activity form two crucial arms of intracellular bacterial killing (*20*). *E. faecalis* can escape intracellular killing by manipulating the endosomal pathway to prevent lysosomal fusion (*15*). We show that MTX-treated macrophages display an enhanced bactericidal activity, rather than an enhanced phagocytosis rate, because of higher levels of expression of lysosomal enzymes including cathepsin D, which has bactericidal activity against both Gram-positive and Gram-negative bacteria (*37*). Inhibition of cathepsin D using pepstatin A ablated MTX-driven killing, whereas inhibiting ROS with MitoTEMPO had minimal effect on MTX-driven killing, suggesting that MTX-induced ROS in macrophages unlikely contributes significantly to intracellular bacterial killing. These results suggest that MTX reprograms macrophages to express elevated levels of lysosomal enzymes and therefore stronger bactericidal activity.

In the more complex system of wound infection in vivo, MTX also augments macrophages to kill bacteria through additional immunoregulatory mechanisms. MTX has previously been shown to inhibit inflammatory responses and induce apoptosis in innate and adaptive immune cells (*35*) and, paradoxically, to induce inflammatory responses and overexpression of M1 markers and NF-κB in macrophages in a dose-dependent manner (*25*, *38*). MTX was also shown to induce inflammatory responses in adult mice following intraperitoneal injection (*38*). In our study, we showed that MTX serves as an immune attractant in infected wounds and is associated with higher levels of proinflammatory cytokines, and the recruited macrophages have higher levels of CD163 and lower expression of CD86. CD163, a macrophage scavenger receptor, is important for bacterial clearance, with CD163^−/−^ mice being highly susceptible to *S. aureus* infection (*39*). By contrast, CD86 can both enhance and impair immune responses to infection, likely depending on the context (*40*). Together, these results show that MTX promotes immunological changes within infected wounds, including recruitment of macrophages with enhanced bactericidal activity, which may contribute to better bacterial clearance.

In summary, we show that MTX has potent antibiotic activity by inducing ROS and DNA damage, resensitizes vancomycin-resistant bacterial strains to vancomycin, and enhances macrophage recruitment to the site of infection and intracellular clearance of bacteria. MTX treatments also enhance wound healing. Our findings support further evaluation of MTX, especially in combination with vancomycin, for treating wound infections by vancomycin-resistant and other bacteria.

### Limitations of this study

Further validation of MTX as an antibiotic and an immunomodulatory agent, incorporating factors of mode of delivery, time of treatment, and dosage will be required to confirm the suitability of this drug as a treatment for bacterial infection. We evaluated the efficacy of MTX in a murine wound excision model of VRE, MRSA, and *P. aeruginosa* infection, but studies in larger animal models and ultimately humans are needed to extend these results. For simplicity, and with an eye toward uncovering mechanistic details, we focused on the effect of MTX upon innate immune cells, and in particular macrophages following a single-dose regime. The effect of multiple doses of MTX and the effect of MTX on other cells (both immune and otherwise), which make up the wound bed milieu, are both important avenues for further investigation. For example, both macrophages and neutrophils were recruited to the site of *E. faecalis* wound infection in mice, but we have not investigated in detail whether and how MTX activates and promotes neutrophil clearance of bacterial infection. Prolonged use of this compound has never been evaluated in infection contexts, but some data suggest that bacteria may be able to metabolize MTX (*67*), which could affect the spectrum of use and could also have implications in the context of polymicrobial infections, if one species metabolically depletes MTX. An important final consideration is that MTX, as a cancer therapeutic, has multiple mechanisms of action and potential broad off-target effects. Moreover, tissues display slow release of MTX (*35*), underscoring that the impact of MTX on tissue healing (following bacterial clearance) will need to be closely monitored. Therefore, treatment using MTX would need to be strategic, for example, as adjuvant therapy or as a last resort in cases of antibiotic-resistant infections, particularly vancomycin-resistant bacteria.

## MATERIALS AND METHODS

### Study design

The study’s objective was to determine the efficacy and the mechanism of action of MTX as an antimicrobial and host-targeted immunotherapy for wound infections. The efficacy of MTX was investigated using a mouse wound excisional model whereby bacterial burden (CFU) was measured 24 hpi. Cells from *E. faecalis* V583–infected mouse wounds were profiled by flow cytometry to identify changes in cell populations between MTX and no treatment. Cytokine levels from treated and nontreated *E. faecalis* V583–infected wounds were also investigated to investigate MTX’s mechanism of action. In addition, in vitro assays with primary and cell lines of murine and human macrophages were designed to identify the individual or combined contributions of MTX and vancomycin in the enhanced bacterial killing observed in vivo. The group sizes for each mouse strain included at least three mice per group with at least two independent experiments to confirm results, which was sufficient to ensure statistical significance, as previously established (*41*, *42*). Experiments were not blinded, and there were no exclusions of data or exclusion criteria to report in this study.

### Ethics statement

All animal experiments were performed with approval from the Institutional Animal Care and Use Committee (IACUC) in Nanyang Technological University (NTU), School of Biological Sciences under protocol ARF-SBS/NIE-A19061.

### Mouse wound excisional model

The procedure for mouse wound infections was modified from a previous study (*15*). Briefly, male C57BL/6 mice (6 to 8 weeks old, 22 to 25 g; NTU, Singapore) were anesthetized with 3% isoflurane. Following dorsal hair trimming, the skin was then disinfected with 70% ethanol before creating a 6-mm full-thickness wound using a biopsy punch (Integra Miltex). Bacteria (1 × 10^7^ CFU) were added to the wound site followed by addition of either 10 μl of PBS or 10 μl of MTX (0.515 μg/ml) immediately. Then, the wound site was sealed with a transparent dressing (Tegaderm 3M). When preventive treatment was tested, PBS or MTX was applied 24 hours before infection, and when cotreatment with vancomycin was performed, intraperitoneal injections of PBS or vancomycin (100 mg/kg in a maximum volume of 100 μl) were performed before the biopsy punch. When multiple treatments with MTX were performed, an 8-mm Finn Chamber on Scanpor was placed around the wound to facilitate removal of the transparent dressing for each treatment without disruption of the underlying bacterial biofilm. In total, five daily treatments of either 10 μl of PBS or 10 μl of MTX (0.515 μg/ml) were applied on the wound. After 24 hpi or 4 days post-infection, mice were euthanized and a 1 cm by 1 cm squared piece of skin surrounding the wound site was excised and collected in sterile PBS. Skin samples were homogenized, and the viable bacteria were enumerated by plating onto BHI plates.

### Histology

Wound tissues were excised as described above and fixed in 4% paraformaldehyde (PFA) in 1× PBS (pH 7.4) for 24 hours at 4°C. Samples were then submerged in 15 and 30% sucrose gradient for 24 hours each, embedded in optimal cutting temperature (OCT) embedding medium (Sakura, California), and frozen in liquid nitrogen. Thin sections (10 μm) were then obtained with a Leica CM1860 ultraviolet cryostat (Leica Biosystems, Ernst-Leitz Strasse, Germany) and stained with H&E (Abcam). Images of H&E-stained sections were acquired using a Zeiss AxioObserver.Z1 inverted wide-field microscope (Carl Zeiss, Göttingen, Germany) fitted with a 5× objective. Generated images by tiling were then manually assembled.

### Bacterial strains and growth conditions

Bacterial strains used in this study are listed in table S4. Bacterial strains were grown using brain heart infusion (BHI) broth and agar (Becton, Dickinson and Company). For experiments performed under anoxic conditions, Oxoid AnaeroGen 3.5L sachet (Thermo Fisher Scientific) was used to create an anoxic atmosphere in the Oxoid chamber. Bacterial strains were streaked from glycerol stocks stored at −80°C, inoculated, and grown overnight statically for 16 to 20 hours in either 10 ml of liquid BHI broth or Dulbecco’s modified Eagle’s medium (DMEM) + 10% fetal bovine serum (FBS) medium. Cells were harvested by centrifugation at 8000 rpm (25°C) for 5 min. The supernatant was discarded, and the pellet was then resuspended in either DMEM + 10% FBS or sterile PBS to an optical density at 600 nm (OD_600_) of 0.7 for VRE, equivalent to 2 × 10^8^ to 3 × 10^8^ CFU.

### Antimicrobial and MIC assays

Bacterial growth assays were carried out in complete DMEM as described previously (*43*). Two microliters of overnight cultures grown in DMEM was added to 200 μl of medium in a 96-well plate with the indicated concentrations of MTX and/or vancomycin. In some assays, MitoTEMPO was also added to a final concentration of 80 μM. The OD_600_ at the zero time point was established. Bacteria were grown statically in 96-well plates at 37°C for up to 24 hours. Final OD_600_ measurements were acquired using a Tecan M200 microplate reader.

### Growth curve assay and kinetic killing assay

Overnight cultures were diluted 1:100 into 96-well plates containing complete DMEM, vehicle [dimethyl sulfoxide (DMSO)], or MTX (0.515 μg/ml; Sigma-Aldrich) and grown at 37°C in a Tecan M200 microplate reader. Every hour, OD_600_ measurements were acquired up to 24 hours. For the kinetic killing assay, overnight cultures of VRE were diluted to a starting CFU equivalent to 10^6^ CFU/ml in 50-ml tubes with 10 ml of DMEM containing MTX (0.515 μg/ml) and/or vancomycin (4 μg/ml) and grown at 37°C. At time points of up to 24 hours after inoculation, 20 μl of culture was removed, serially diluted in sterile PBS, and spot-plated onto BHI agar for CFU calculation.

### MTX uptake assay

VRE overnight cultures were diluted 10-fold into 10 ml of complete DMEM in 50-ml tubes containing either MTX (0.515 μg/ml) or MTX (0.515 μg/ml) and vancomycin (4 μg/ml). After 6 hours of growth at 37°C, 1-ml aliquots were removed from each sample and washed three times with PBS. Next, 200 μl of each test sample was transferred to black-walled 96-well plates to measure MTX fluorescence (excitation = 610 nm, emission = 685 nm) by using a Tecan M200 microplate reader. MTX fluorescence measurement was acquired as established in (*30*), and MTX levels were normalized to OD_600_ to account for differences in *E. faecalis* V583 growth.

### Bacterial cell permeability assay

VRE overnight cultures were diluted 10-fold into 10 ml of complete DMEM in 50-ml tubes containing either MTX (0.515 μg/ml), vancomycin (4 μg/ml), or MTX (0.515 μg/ml) and vancomycin (4 μg/ml). After 6 hours of growth at 37°C, 1 ml of aliquot was removed and washed three times with PBS before addition of PI to a final concentration of 20 μM. PI uptake was used as a way to estimate permeability as established previously (*44*). Cells were then fixed in 4% PFA for 15 min before PI fluorescence analysis using the BD LSRFortessa X-20 Cell Analyzer (Becton Dickinson). In addition, epifluorescence microscopy VRE images after permeability assay were acquired using a 63× oil objective (Zeiss) fitted onto a Zeiss AxioObserver.Z1 inverted wide-field microscope (Carl Zeiss, Göttingen, Germany). Acquired images were visually analyzed using ImageJ.

### Liquid chromatography–mass spectrophotometry

Sample extraction and measurement followed the published reports with modifications (*45*, *46*). Overnight cultures of VRE were diluted in a ratio of 1:10 in DMEM + 10% FBS and statically incubated in DMEM alone, 1 μM MTX, and/or vancomycin (4 μg/ml) at 37°C. Bacteria were harvested at 1 hour, and CFU was determined by plating serial dilutions on BHI agar medium. The cell-free supernatant was collected by filtration through a 0.22-μm filter. MTX was extracted by adding ice-cold 50:50 acetonitrile/methanol. After vortexing, the mixture was centrifuged and the supernatant was collected and evaporated to dryness in a vacuum evaporator. The dry extracts were redissolved in 50:50 water/methanol for liquid chromatography–mass spectrometry (LC-MS) analysis. The calibration curve was prepared by spiking MTX standard solutions into blank medium, which proceeded as described above. LC-MS analysis was performed with Agilent 1290 ultrahigh pressure liquid chromatography system coupled to electrospray ionization with iFunnel Technology on a 6490 triple quadrupole mass spectrometer. Chromatographic separation was achieved by using a Waters Atlantis T3 column with mobile phases (A) 0.1% formic acid in water and (B) 0.1% formic acid in methanol. Electrospray ionization was performed in positive ion mode, and MTX was quantified in multiple reaction monitoring mode with the transitions of mass/charge ratio (*m/z*) 445 > 88 and *m/z* 445 > 70. Data acquisition and processing were performed using MassHunter software (Agilent Technologies). The intracellular MTX was calculated as follows: [MTX]_drug-only control_ − [MTX]_filtrate_.

### DNA damage measurement

VRE overnight cultures were diluted 10-fold into 10 ml of complete DMEM in 50-ml tubes containing MTX (0.515 μg/ml) and vancomycin (4 μg/ml), separately and in combination, and the positive control H_2_O_2_ (0.1 mM). After 6 hours of growth at 37°C, 1 ml of aliquot was removed. Bacterial cells were pelleted by centrifugation (8000 rpm, 5 min), and the supernatant was used to determine the levels of 8-OHdG using a DNA Damage Competitive ELISA (enzyme-linked immunosorbent assay) assay as per the manufacturer’s instructions and previous study (*47*).

### Bacterial ROS quantification

This assay was adapted from (*43*). *E. faecalis* V583 overnight cultures were diluted 10-fold into 200 μl of complete DMEM in black-walled 96-well plates containing 50 μM DHR123 (Thermo Fisher Scientific), vehicle (DMSO), MTX (0.515 μg/ml), and/or vancomycin (4 μg/ml). Plates were incubated with no shaking at 37°C for 6 hours. At the end, the optical density was measured at 600 nm to determine bacterial growth, and DHR123 fluorescence (excitation = 507 nm, emission = 529 nm) was measured using a Tecan M200 microplate reader to determine cellular ROS levels. ROS levels were normalized to OD_600_ to account for differences in VRE growth.

### In vitro evolution of *E. faecalis* to MTX resistance

The protocol was adapted from a previously published in vitro evolution experiment done in *E. faecalis* V583 (*48*). Sixteen starting colonies were picked from an overnight grown plate and grown in DMEM + 10% FBS overnight for performing parallel lines of evolution experiment. Dilutions (10×) of overnight bacterial cultures of each strain were made in DMEM + 10% FBS containing MTX from a starting sub-MIC concentration of 0.258 μg/ml and incubated at 37°C at static conditions for 22 to 26 hours. Cultures of every evolution line were examined for visible bacterial growth. Bacterial cultures were then diluted again 10× into fresh MTX-containing medium at higher concentration. This was repeated until MTX of 2.84 μg/ml was achieved. Bacterial cultures were then further passaged two more times in the highest concentration, before genomic DNA extraction using the Wizard Genomic DNA Purification Kit (Promega, USA). Each of the 16 bacterial cultures was also further evaluated for MIC method, as described above, to validate their susceptibility to higher concentrations of MTX. Evolved strains were also subjected to whole-genome sequencing. Raw reads were imported into CLC Genomics Workbench 8.0 (QIAGEN), followed by quality trimming to remove bad quality reads. The trimmed reads were then mapped to the reference genome before the Basic Variant Detection module was used to detect for mutations using the default parameters.

### Strain construction

To construct the V583DEAD (*dead/deah* gene WT copy) and MTX^R^V583DEAD (*dead/deah* mutated gene copy) complementation plasmid 1, primers 1 and 2 (tables S5 and S6) were designed with Xho I restriction sites. These primers flank the gene of interest and were used to amplify the DNA sequence from the isolated genomic DNA of the WT and MTX^R^ strains. In-Fusion cloning (TaKaRa Bio) was performed using primers 1 and 2 with at least 15–base pair complementary sequence for ligation into vector pGCP123 (*49*), which was also digested with the same restriction enzyme. The pGCP123::V583DEAD and pGCP123::MTX^R^V583DEAD plasmid was generated in *E. coli* DH5α, verified by sequencing, and transformed into *E. faecalis* as described previously (*49*). Similarly, the *van* operon without the two-component system *van*RS from the VRE strain was cloned using In-Fusion cloning (Takara Bio). Plasmid 2 was used, and primers 3 and 4 (tables S5 and S6) were designed to incorporate the restriction sites Xho I and Bam HI. The genes EF_RS05155, EF_RS07835, and EF_RS09020 from both VRE and VRE MTX^R^ strains were also cloned using In-Fusion cloning (TaKaRa Bio) with plasmid 1 and primers 5 to 14 (tables S5 and S6). Inverse PCR was used to amplify the vector pGCP123, and vector overlapping regions were incorporated in the primers to amplify the genes instead of restriction sites.

### Flow cytometry

Flow cytometry was performed as described in (*14*) with some modifications. Excised skin samples were placed in 1.5 ml of Eppendorf tubes containing liberase (2.5 U/ml) prepared in DMEM with gentamicin and penicillin G (500 μg/ml) (Sigma-Aldrich). The mixture was then transferred into six-well plates and incubated for 1 hour at 37°C in a 5% CO_2_ humidified atmosphere with constant agitation. Dissociated cells were then passed through a 70-μm cell strainer to remove undigested tissues and were spun down at 1350 rpm for 5 min at 4°C. The enzymatic solution was then aspirated, and cells were blocked in 500 μl of fluorescence-activated cell sorting (FACS) buffer [2% FBS and 0.2 mM EDTA in PBS (Gibco; Thermo Fisher Scientific)]. Cells (10^7^ per sample) were then incubated with 10 μl of Fc-blocker (anti-CD16/CD32 antibody; BioLegend) for 30 min, followed by incubation with an anti-mouse CD45, CD11b, and Ly6G (neutrophils), or CD45, CD11b, and F4/80 (macrophages) plus CD86 and MHCII or CD163 and CD206 marker-conjugated antibodies (BioLegend) (1:100 dilution) for 30 min at room temperature. Cells were then centrifuged at 500*g* for 5 min at 4°C and washed in FACS buffer. Cells were fixed in 4% PFA for 15 min at 4°C, before final wash in FACS buffer and final resuspension in this buffer. Following which, cells were analyzed using the BD LSRFortessa X-20 Cell Analyzer (Becton Dickinson). Compensation was done using the AbC Total Antibody Compensation Bead Kit (Thermo Fisher Scientific) as per the manufacturer’s instructions. To evaluate MTX cytotoxicity in RAW264.7 cells, cells were analyzed using the BD LSRFortessa X-20 Cell Analyzer after being stained with PI (final concentration of 20 μM) for 30 min on ice.

### Cytokine analysis

Homogenized wound samples were stored at −80°C until use. Samples were thawed on ice and centrifuged for 5 min at 500*g* to remove cell debris. Supernatants were used to perform ELISA to quantify the levels of IL-1β (Thermo Fisher Scientific), IL-6 (BioLegend), TNF-α (BioLegend), IFN-γ (BioLegend), and TGF-β1 (Thermo Fisher Scientific), according to the manufacturer’s instructions.

### Human blood-derived macrophages and cell lines

Isolated peripheral blood primary human monocytes were purchased from STEMCELL Technologies. For in vitro differentiation of monocytes into human macrophages, isolated monocytes were cultured in complete RMPI 1640 supplemented with 10% heat-inactivated FBS (PAA, GE Healthcare), 2 mM l-glutamine (Corning), and 1% penicillin-streptomycin solution (Gibco; Thermo Fisher Scientific) in the presence of recombinant human macrophage colony-stimulating factor (M-CSF) (50 ng/ml) (BioLegend) for 7 days. The RAW264.7 murine macrophage-like cell line (InvivoGen) and the THP-1 monocytic cells derived from an acute monocytic leukemia patient cell line (American Type Culture Collection) were cultured at 37°C in a 5% CO_2_ humidified atmosphere. All cells were grown and maintained in DMEM (Gibco; Thermo Fisher Scientific) with 10% heat-inactivated FBS (PAA, GE Healthcare),and 100 U of penicillin-streptomycin (Gibco; Thermo Fisher Scientific). The culture medium was replaced every 3 days, and upon reaching 80% confluency, cultures were passaged. RAW264.7 cell passaging was achieved by gentle cell scraping and seeding cells at a density of 3 × 10^6^ cells per T75 flask (Nunc; Thermo Fisher Scientific).

### Mouse BMDM and neutrophil isolation

BMDMs were prepared as described previously (*50*). Briefly, fresh bone marrow cells were isolated from mice, plated in complete RPMI with recombinant M-CSF (50 ng/ml) (BioLegend), and cultured for 6 days with medium change every 3 days. A MojoSort mouse neutrophil isolation kit (BioLegend) was used to isolate murine neutrophils as per the manufacturer’s instructions.

### Intracellular infection assay

Intracellular infection assays were performed as described in (*14*) with some modifications. Cells were seeded at a density of 10^6^ cells per well or 8 × 10^5^ cells per well in a 6-well or 96-well tissue culture plate (Nunc; Thermo Fisher Scientific), respectively, and allowed to attach overnight at 37°C in a 5% CO_2_ humidified atmosphere. Cells were infected at a multiplicity of infection (MOI) of 10 for up to 3 hours. Following infection, the medium was aspirated, and the cells were washed three times in PBS and incubated with gentamicin (150 μg/ml) (Sigma-Aldrich) and penicillin G (50 μg/ml) (Sigma-Aldrich) to kill extracellular bacteria and MTX (0.515 μg/ml), or varying concentrations of vancomycin (0.06 to 75 μg/ml) (Sigma-Aldrich) and MTX (0.515 μg/ml), in complete DMEM for 18 to 24 hours to selectively kill extracellular bacteria. The antibiotic-containing medium was then removed, and the cells were washed three times in PBS before addition of 2% Triton X-100 (Sigma-Aldrich) PBS solution to lyse the cells for enumeration of the intracellular bacteria. Variations of this assay included pretreatment of mammalian cells, before bacterial infection, with MTX (0.515 μg/ml) followed by antibiotic treatment only or cotreatment of cells at the time of infection with either MitoTEMPO (80 μM) (Sigma-Aldrich) or pepstatin A (10 μg/ml) (Sigma-Aldrich).

### NF-κB reporter assay

This assay was performed as described in (*13*) using RAW-blue cells (InvivoGen). After treatment of RAW264.7 cells for 16 hours with MTX (0.515 μg/ml) or lipopolysaccharide (100 ng/ml) and IFN-γ (50 ng/ml) or IL-4 (10 ng/ml) and IL-13 (10 ng/ml), 20 μl of supernatant was added to 180 μl of Quanti-Blue reagent (InvivoGen) and incubated overnight at 37°C. Secreted alkaline phosphatase (SEAP) levels were determined at 640 nm by using a Tecan M200 microplate reader.

### Lactate dehydrogenase cell viability assay

As described before (*13*), after intracellular infection assays, culture supernatants were collected from each well to measure lactate dehydrogenase (LDH) release using an LDH cytotoxicity assay (Clontech) according to the manufacturer’s instructions. Background LDH activity was determined using mock (PBS)–treated RAW264.7 cells. Maximal LDH activity was determined by lysing cells with 1% Triton X-100. The percentage of cytotoxicity was calculated as follows: % cytotoxicity = [(sample absorbance − background absorbance)/(maximal absorbance − background absorbance)] × 100.

### Mammalian cell ROS quantification

Mammalian cells were seeded at a density of 8 × 10^5^ in a 96-well tissue culture plate (Black Nunc; Thermo Fisher Scientific) and allowed to attach overnight at 37°C in a 5% CO_2_ humidified atmosphere. Fluorescein (Abcam) was added to each well to a final concentration of 120 nM followed by addition of the positive control (H_2_O_2,_ 1 mM), vehicle (DMSO), and MTX (0.515 μg/ml). Cells were then either infected at an MOI of 10 for up to 6 hours or left uninfected. Plates were incubated with no shaking at 37°C. At the end, the fluorescence (excitation = 490 nm, emission = 525 nm) was measured using a Tecan M200 microplate reader to determine cellular ROS levels.

### RNA isolation and qRT-PCR

To quantify the levels of RNA transcripts, total RNA was extracted from nontreated, DMSO-treated, or MTX-treated RAW264.7 cells with the RNeasy MinElute Kit (QIAGEN) and reverse-transcribed using SuperScript III First-Strand Synthesis SuperMix (Thermo Fisher Scientific), followed by amplification with KAPA SYBR Fast (Kapa Biosystems) with specific primers (table S6) and detected by StepOnePlus Real-Time PCR machine (Applied Biosystems). Relative quantification of gene expression was performed using the comparative *C*_T_ method (*51*), where the *C*_T_ values were normalized with the housekeeping gene glyceraldehyde phosphate dehydrogenase (*GAPDH*) for comparison. Similarly, total RNA was extracted from bacteria without treatment or treated with DMSO, MTX, vancomycin, or MTX plus vancomycin for 6 hours with TRIzol (Thermo Fisher Scientific) followed by the RNeasy MinElute Kit (QIAGEN) and reverse-transcribed using SuperScript III First-Strand Synthesis SuperMix (Thermo Fisher Scientific). Relative levels of gene expression were quantified using the comparative *C*_T_ method (*51*), where the *C*_T_ values were normalized with the housekeeping gene *rpoB* for comparison.

### Immunoblotting

Whole-cell lysates were prepared by adding 488 μl of radioimmunoprecipitation assay (RIPA) buffer [50 mM tris-HCl (pH 8.0), 1% Triton X-100, 0.5% sodium deoxycholate, 0.1% SDS, and 150 mM NaCl] to the wells after intracellular infection assays, where cells were scraped and kept in RIPA buffer for 30 min at 4°C. Before the addition of 74.5 μl of 1 M dithiothreitol and 187.5 μl of NuPAGE LDS sample buffer (4×) (Thermo Fisher Scientific), cells were further mechanically disrupted by passing the lysate through a 26-gauge size needle. Samples were then heated to 95°C for 5 min. Fifteen microliters of cell lysate proteins was then separated in a 4 to 12% (w/v) NuPAGE Bis-Tris protein gel and transferred to polyvinylidene difluoride membranes. Membranes were incubated with tris-buffered saline [50 mM tris and 150 mM NaCl (pH 7.5)] containing 0.1% (v/v) Tween 20 (TBST) and 5% (w/v) bovine serum albumin (BSA) for 1 hour at room temperature. Membranes were incubated with 1:1000 for rabbit α-cathepsin D (Cell Signaling Technology) or 1:1000 for rabbit α-GAPDH (Cell Signaling Technology) in TBST containing 1% (w/v) BSA overnight at 4°C. Membranes were washed for 60 min with TBST at room temperature and then incubated for 2 hours at room temperature with goat anti-rabbit (H+L) horseradish peroxidase–linked secondary antibody (Invitrogen). After incubation, membranes were washed with TBST for 30 min, and specific protein bands were detected by chemiluminescence using a SuperSignal West Femto maximum sensitivity substrate (Thermo Fisher Scientific). Band intensities were quantified relatively to the lane’s loading control using ImageJ (*52*).

### Phagocytosis assay

*E. faecalis* V583 cells were fixed with 4% PFA for 15 min and washed thrice with PBS, before labeling with the membrane-permeant DNA dye Syto9 (Thermo Fisher Scientific). Bacterial cells were then washed thrice with PBS and resuspended in DMEM + 10% FBS. RAW264.7 cells were infected with MOI of 10 of Syto9-labeled bacterial cells and incubated for 1 hour at 37°C and 5% CO_2_. Following supernatant removal, infected cells were harvested and resuspended in PBS. The fluorescence of bacteria either free in the medium or attached to the RAW264.7 cell membranes was quenched with a final concentration of 0.01% trypan blue. As trypan blue cannot enter viable eukaryotic cells, the unquenched fluorescence reflected the bacterial cells that were internalized in viable RAW264.7 cells. After staining, cells were immediately run through the flow cytometer. All data were collected using the BD LSRFortessa X-20 Cell Analyzer and analyzed with FlowJo V10.8.1 (BD Biosciences, USA). The samples were initially gated side scatter area (SSC-A) by forward scatter area (FSC-A) to select the RAW264.7 populations. The RAW264.7 population was subsequently gated forward scatter width (FSC-W) by SSC-A to remove doublet populations. The resulting single-cell population was then assessed for Syto9 fluorescent marker.

### Fluorescence staining

RAW264.7 cells were seeded at 2 × 10^5^ cells per well in a 24-well plate with 10-mm coverslips and allowed to attach overnight at 37°C and 5% CO_2_. Infection with Syto9 fluorescent-labeled *E. faecalis* V583 was performed with MOI of 10 for 1 hour. The coverslips seeded with cells were then fixed with 4% PFA at 4°C for 15 min, permeabilized with 0.1% Triton X-100 for 15 min at room temperature, and washed thrice in PBS. Cells were then blocked with PBS supplemented with 0.1% saponin and 2% BSA. For actin labeling, the phalloidin–Alexa Fluor 555 conjugate (Thermo Fisher Scientific, USA) was diluted 1:40 in PBS and incubated for 1 hour. Coverslips were then washed three times in PBS with 0.1% saponin. They were then subjected to a final wash with PBS, thrice. Last, the coverslips were mounted with SlowFade Diamond Antifade (Thermo Fisher Scientific) and sealed. Confocal images were then acquired on a 63×/numerical aperture (NA) 1.4, Plan Apochromat oil objective fitted onto Elyra PS.1 with LSM 780 confocal unit (Carl Zeiss) using the Zeiss Zen Black 2012 FP2 software suite. Laser power and gain were kept constant between experiments. Z-stacked images were processed using Zen 2.1 (Carl Zeiss). Acquired images were visually analyzed using ImageJ (*52*).

### Statistical analysis

Statistical analysis was done using Prism 9.2.0 (GraphPad, San Diego, CA). We used nonparametric Mann-Whitney test to compare ranks, and one-way analysis of variance (ANOVA) with appropriate post-tests, as indicated in the figure legend for each figure, to analyze experimental data comprising three independent biological replicates, where each data point is typically the average of a minimum two technical replicates (unless otherwise noted). In all cases, a *P* value of ≤0.05 was considered statistically significant.

## Supplementary Materials

### This PDF file includes:

Figs. S1 to S13 Tables S1 to S6 References

## Notes

View/request a protocol for this paper from *Bio-protocol*.
