# Supplementary material for: Mitoxantrone targets both host and bacteria to overcome vancomycin resistance in Enterococcus faecalis
Source: Sci Adv. 2023 Feb 22;9(8):eadd9280. doi: 10.1126/sciadv.add9280 (PMC9946351; doi:10.1126/sciadv.add9280)
Supplement: Supplementary file 1 — Figs. S1 to S13 Tables S1 to S6 References [file sciadv.add9280_sm.pdf]

Science Advances
AAAS

Supplementary Materials for

Mitoxantrone targets both host and bacteria to overcome vancomycin resistance in *Enterococcus faecalis*

Ronni A. G. da Silva *et al.*

Corresponding author: Jianzhu Chen, jchen@mit.edu; Kimberly A. Kline, kimberly.kline@unige.ch

Sci. Adv. **9**, eadd9280 (2023)
DOI: 10.1126/sciadv.add9280

This PDF file includes:

Figs. S1 to S13
Tables S1 to S6
References

Supplementary Figures and Tables:

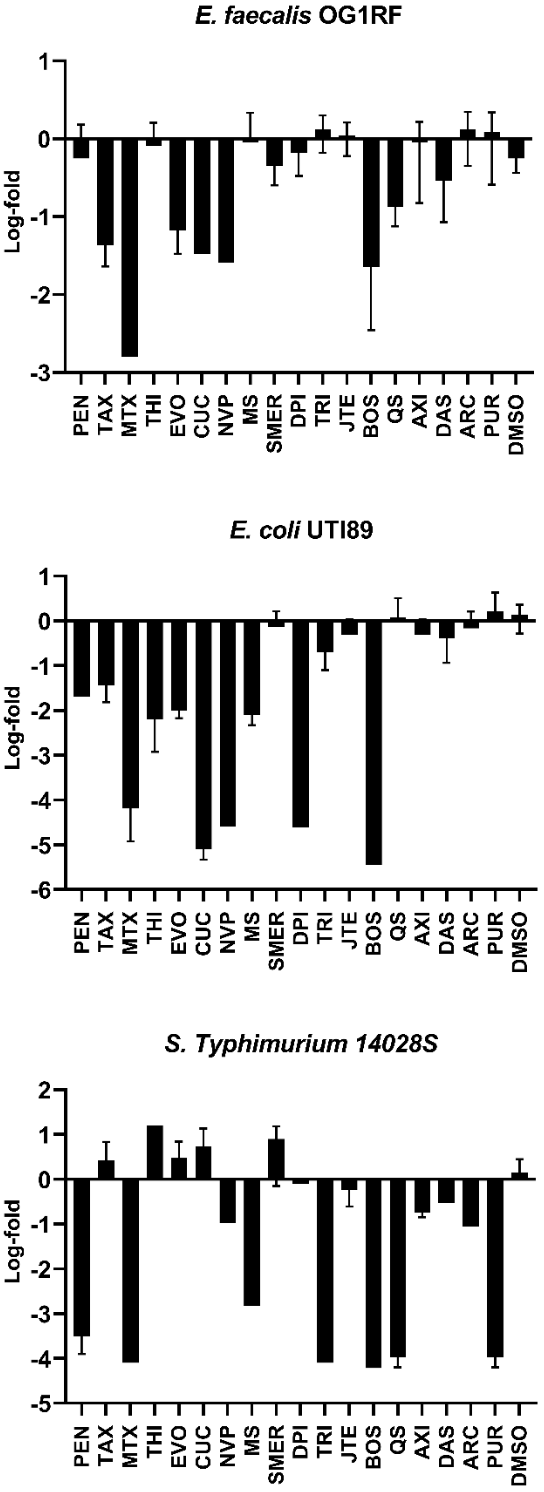

Fig. S1 – MTX induces intracellular killing of bacteria.

Raw264.7 cells were infected with the indicated bacterial species for 3 h, followed by 1 h of antibiotic treatment to kill extracellular bacteria. After removal of the first antibiotic medium, and wash, a new solution with antibiotics was added together with

18 different compounds. Viable intracellular CFU were enumerated after 18 h incubation. The log-fold change was calculated with reference to the intracellular CFU of infected cells that were not treated with any compound. MTX-treated cells had lower intracellular CFU regardless of the bacterial species tested.

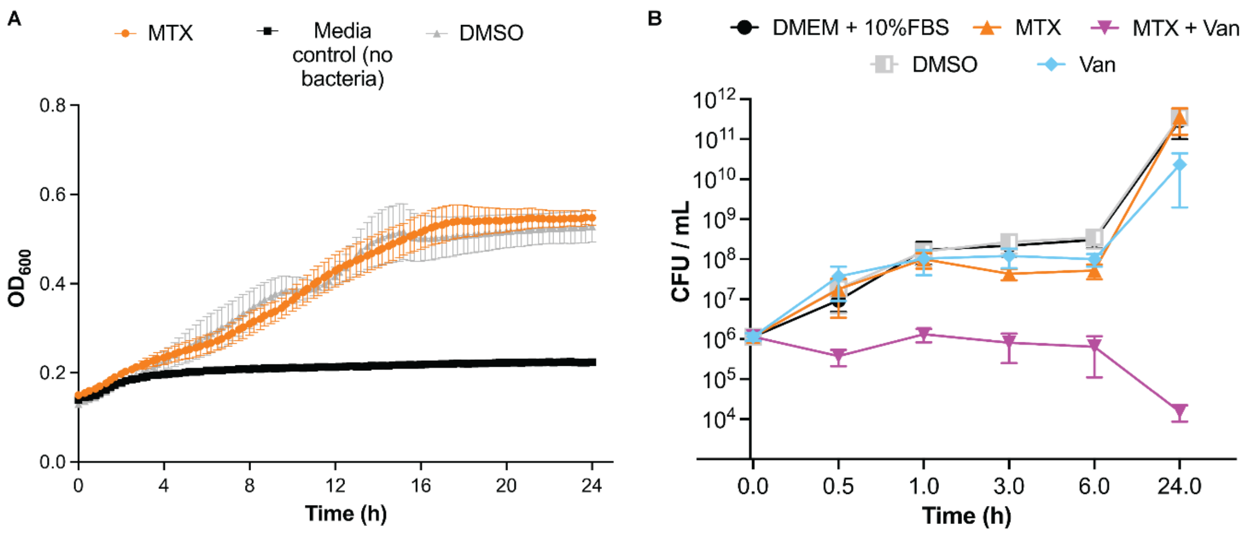

Fig. S2- Low dose of MTX in presence of low dose of vancomycin inhibits VRE growth.

A. Growth curve of VRE in the presence of MTX (0.515 µg/mL) in DMEM for 24 h**. B.** VRE growth curve by CFU counting. In a 10 mL tube, a bacterial starting culture of 10^6 CFU/mL was incubated with MTX, vancomycin, separately or in combination, and vehicle (DMSO). At different time points, serial dilutions were performed in a 96-well plate and spotted on a BHI plate to establish the CFU/mL. **A and B**. Data (mean ± SEM) are a combination of at least three independent experiments.

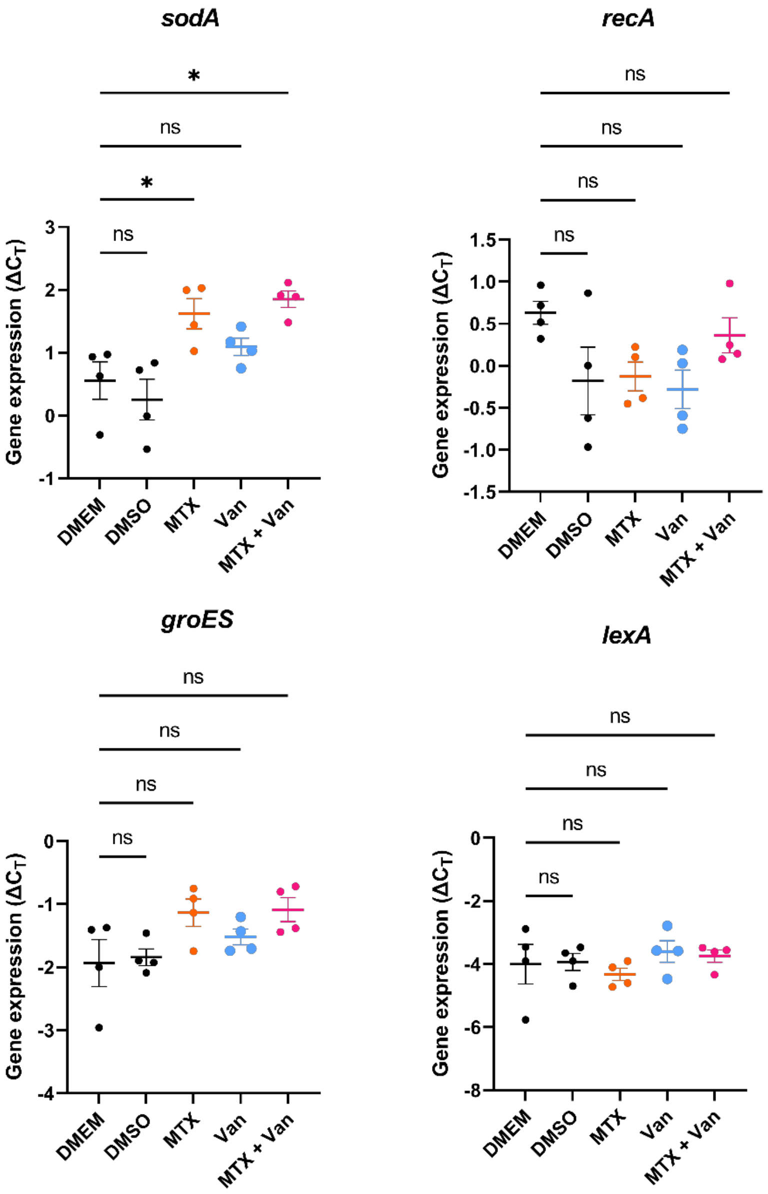

Fig. S3- Bacterial stress response genes expression is affected by MTX treatment.

qRT-PCR analysis of transcript levels (ΔC_T) of stress response genes in VRE without treatment (DMEM), or treated with DMSO, MTX (0.515 μg/mL) or vancomycin (4 μg/mL), or MTX plus vancomycin for 6 h. Each dot represents one biological replicate. Statistical analysis was performed using ordinary one-way ANOVA, followed by Tukey's multiple comparison test, NS $p > 0.05$ and $^*p \leq 0.05$.

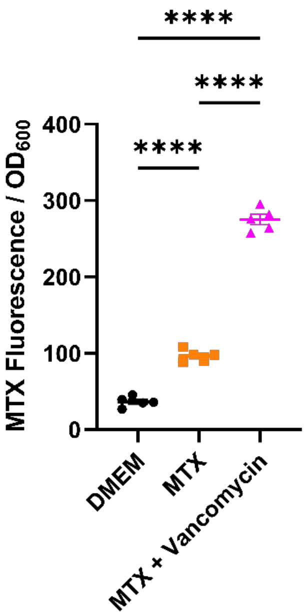

Fig. S4- MTX uptake is not affected by anoxic conditions.

MTX uptake by VRE after 6 h treatment with MTX (0.515 µg/mL) alone and in combination with vancomycin (4 µg/mL) incubated in anoxic conditions**.** Each dot represents one independent experiment. Statistical analysis was performed using ordinary one-way ANOVA, followed by Tukey's multiple comparison test, ****$p \leq 0.0001$.

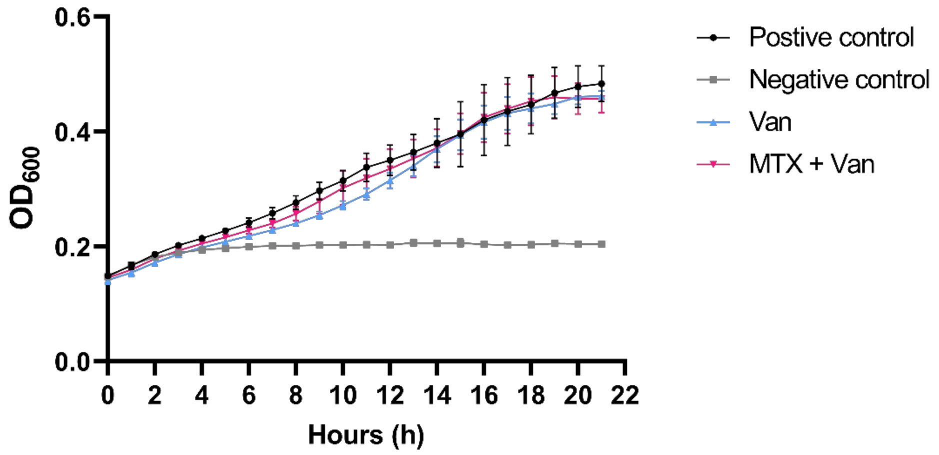

Fig. S5- VRE MTX^R growth is not affected by vancomycin alone or vancomycin plus MTX.

Growth curve of VRE MTX^R in presence of vancomycin (4 µg/mL) or vancomycin plus MTX (0.515 µg/mL) in DMEM for 21 h. Positive control is VRE MTX^R growth in DMEM alone and negative control is DMEM alone. Data (mean ± SEM) are a combination of two independent experiments.

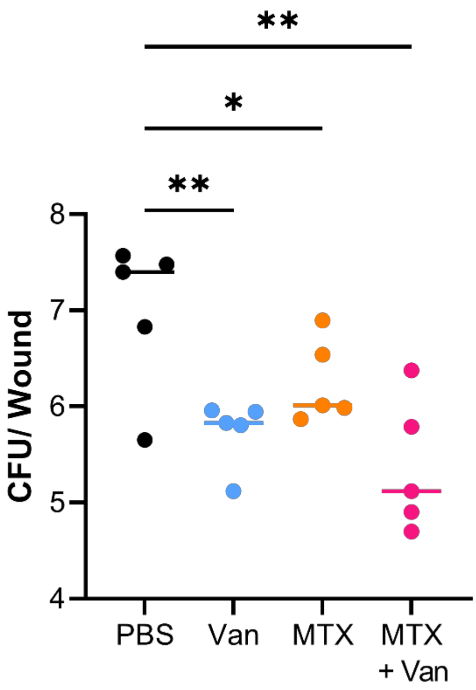

Fig. S6- MTX and vancomycin co-treatment increases killing of the VRE MTX^R strain during wound infection.

Data are from two independent experiments with 2-3 mice per experiment. Each symbol represents one mouse with median indicated by the horizontal line. Statistical analysis was performed using Kruskal Wallis test with uncorrected Dunn's post-test, *p ≤ 0.05; **p ≤ 0.01.

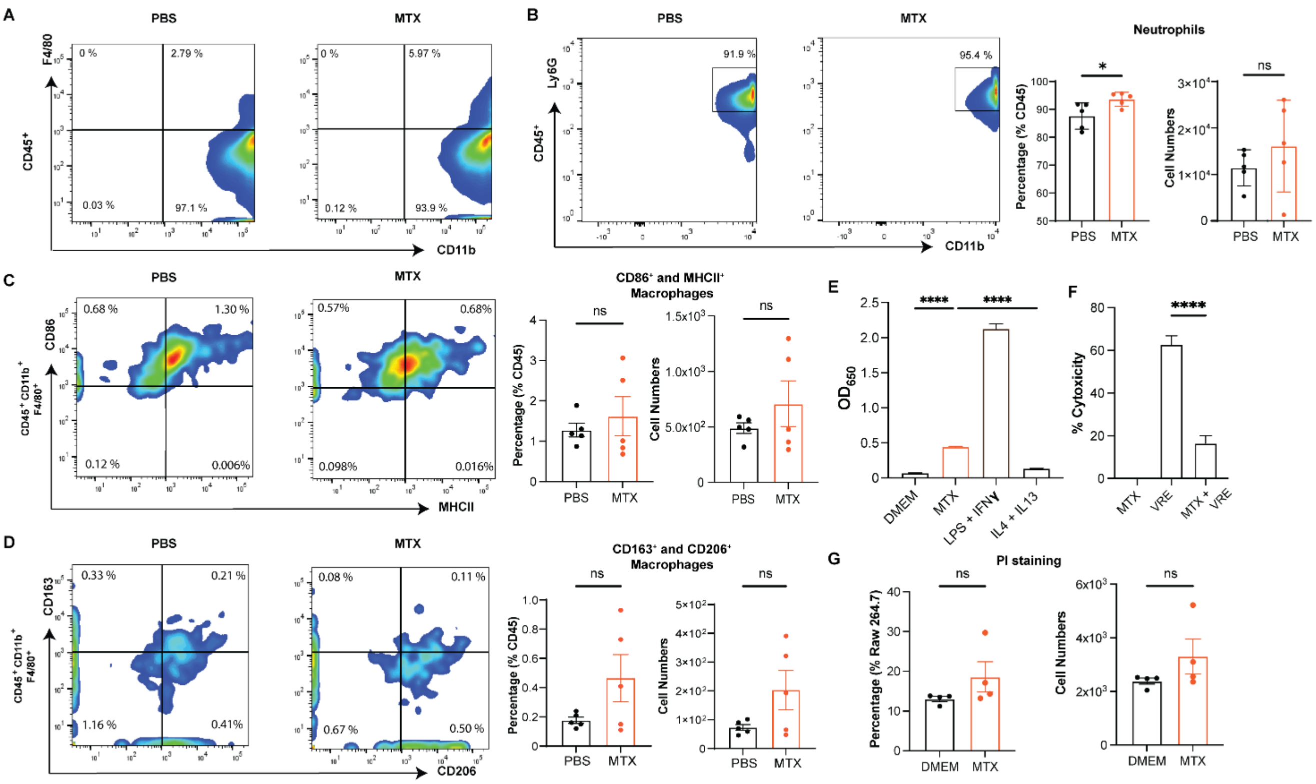

Fig. S7- MTX treatment does not affect macrophage polarization but influences NF-κB activity.

VRE Infected mouse wounds were treated for 24 h with either PBS or a single dose of MTX (0.515 μg/mL, 10 μL on the wound). **A.** Representative flow cytometry of macrophages ($CD45^+$ $CD11b^+$ $F4/80^+$) from infected wounds treated with PBS or MTX. The number indicate percentages of cells within the gated areas. **B.** Representative flow cytometry of neutrophils ($CD45^+$ $CD11b^+$ $Ly6G^+$) from infected wounds treated with PBS or MTX. The number indicate percentages of cells within the gated areas. Percentage and absolute numbers of neutrophils recovered from infected wounds treated with PBS or MTX. Each dot represents one mouse. **C.** Representative flow cytometry of macrophages ($CD45^+$ $CD11b^+$ $F4/80^+$ $CD86^+$ $MHCII^+$) from infected wounds treated with PBS or MTX. The number indicate percentages of cells within the gated areas. Percentage and absolute numbers of $CD86^+$ $MHCII^+$ macrophages recovered from infected wounds treated with PBS or MTX. Each dot represents one mouse. **D.** Representative flow cytometry of macrophages ($CD45^+$ $CD11b^+$ $F4/80^+$ $CD163^+$ $CD206^+$) from infected wounds treated with PBS or MTX. The number indicate percentages of cells within the gated areas. Percentage and absolute numbers of

CD163$^+$ CD206$^+$ macrophages recovered from infected wounds treated with PBS or MTX. Each dot represents one mouse. **B-D**. Statistical analysis was performed using unpaired T-test with Welch's corrections, NS $p > 0.05$. **E.** NF-κB-driven SEAP reporter activity. RAW267.4 macrophages were untreated or treated with MTX or LPS (100 ng/mL) and IFN-γ (50 ng/mL) or IL-4 (10 ng/mL) and IL-13 (10 ng/mL) 16 h prior to measurement of NF-κB-driven SEAP reporter activity. **F.** LDH activity after intracellular infection assay with MTX. RAW267.4 macrophages were infected or not with VRE MOI 10 for 3 h and then left untreated or treated with MTX 16 h in presence of antibiotic treatment prior to measurement cytotoxicity (LDH activity). While supernatant of untreated cells were considered the background noise and were subtracted from the test values, cells lysed with Triton X-100 were considered 100 %. **E-F.** Data (mean ± SEM) are a summary of at least three independent experiments. Statistical analysis was performed using ordinary one-way ANOVA, followed by Tukey's multiple comparison test, NS $p > 0.05$; *$p \leq 0.05$; **$p \leq 0.01$; ***$p \leq 0.001$ and ****$p \leq 0.0001$. **G.** Percentage and absolute numbers of PI staining of RAW267.4 macrophages untreated or treated with MTX for 16 h prior to measurement. Data (mean ± SEM) are a summary of at least three independent experiments. Statistical analysis was performed using unpaired T-test with Welch's corrections, NS $p > 0.05$.

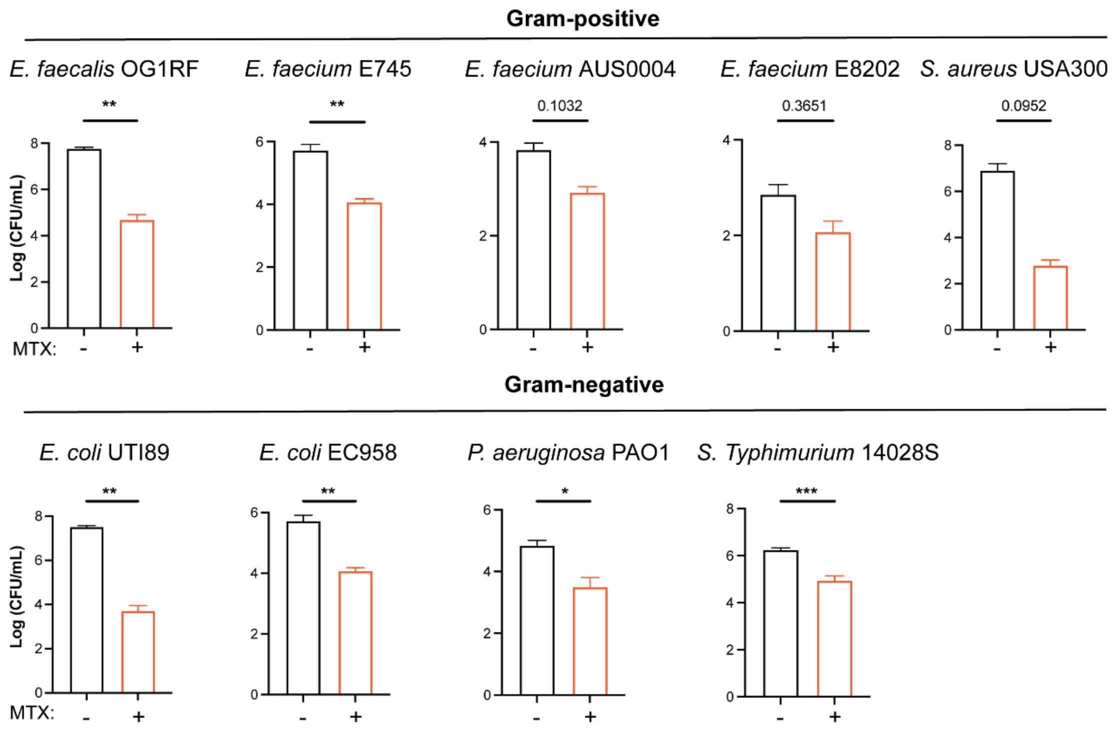

Fig. S8- MTX as antimicrobial against multiple bacterial species *in vitro*.

A. Comparison of CFU counts of different Gram-positive and Gram-negative bacterial species in RAW264.7 in presence or absence of MTX. Data (mean ± SEM) are summary of three independent experiments. Statistical analysis was performed using the non-parametric Mann-Whitney Test to compare ranks, NS p value is shown; *p ≤ 0.05; **p ≤ 0.01; ***p ≤ 0.001.

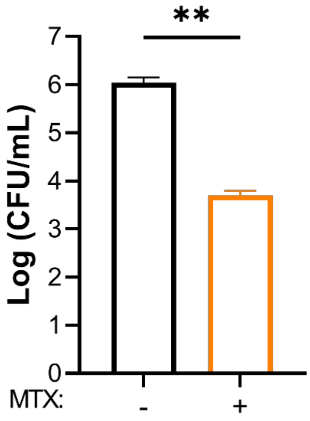

Fig. S9- MTX enhances neutrophil killing of bacteria.

Comparison of VRE CFU counts in murine neutrophils in the presence or absence of MTX. Data (mean ± SEM) are summary of at least three independent experiments. Statistical analysis was performed using unpaired T-test with Welch's corrections, **p ≤ 0.01.

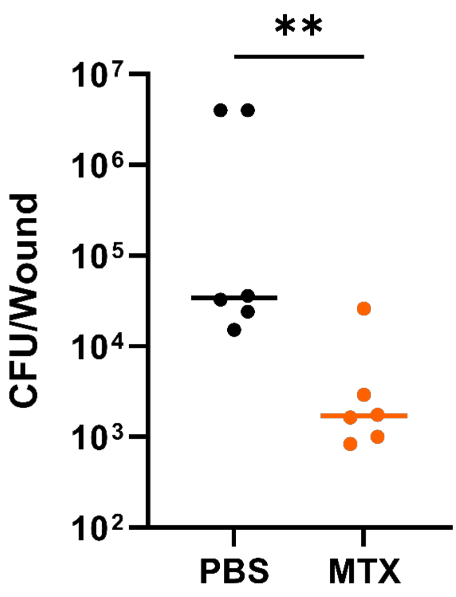

Fig. S10- Pre-treatment of wounds with MTX prior to infection reduces bacterial load.

Comparison of VRE CFU per wound 24 h after infection. Treatments with PBS or MTX were performed 24 h before infection. Data are from two independent experiments with 3 mice per experiment. Each symbol represents one mouse with median indicated by the horizontal line. Statistical analysis was performed using Mann-Whitney test to compare ranks, $**p \leq 0.01$.

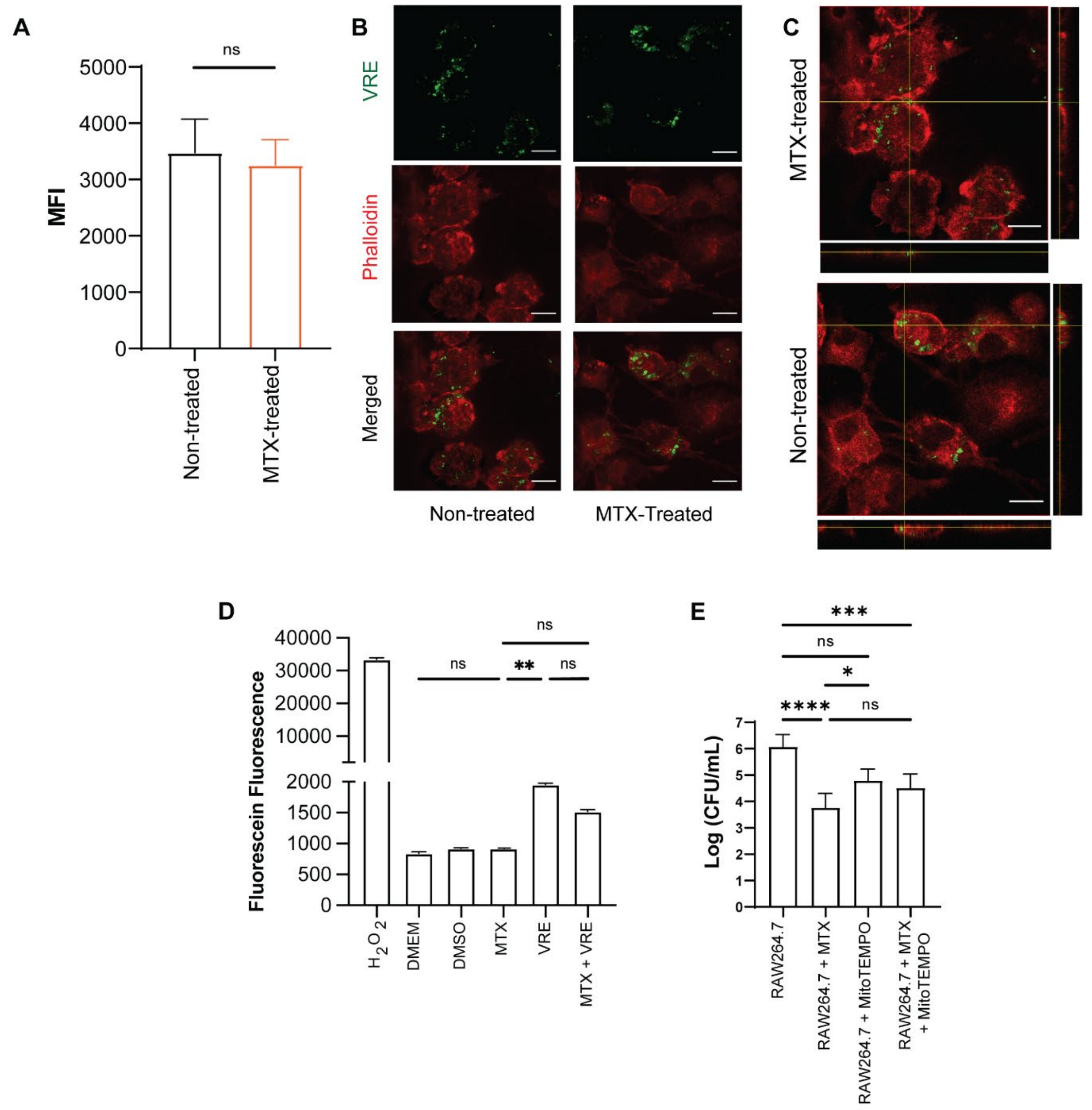

Fig. S11- MTX does not induce phagocytosis nor ROS in RAW264.7. A - C. Phagocytosis assay. MTX pre-treated RAW264.7 macrophages were infected for 1 h with SYTO9-labelled VRE, quenched with Trypan Blue and then run through a flow cytometer for green fluorescence measurement. **A.** Mean Fluorescence Intensity (MFI). Data (mean ± SEM) are a summary of at least three independent experiments. Statistical analysis was performed using unpaired T-test with Welch's corrections, NS $p > 0.05$. **B and C.** Representative CLSM images and orthogonal views of matching samples that were also stained with phalloidin ultra-cellular structure visualization. Scale bar: 20 µm. **D.** ROS levels of RAW264.7 macrophages untreated (DMEM) or treated with H_2O_2 (1 mM, positive control), DMSO, or MTX and infected or not with

VRE for 6h. **E.** Comparison of VRE CFU counts in RAW264.7 in presence or absence of MTX and/or MitoTEMPO (80 μM). **D-E.** Data (mean ± SEM) are a summary of at least three independent experiments. Statistical analysis was performed using ordinary one-way ANOVA, followed by Tukey's multiple comparison test, NS p > 0.05; *p ≤ 0.05; **p ≤ 0.01; ***p ≤ 0.001 and ****p ≤ 0.0001.

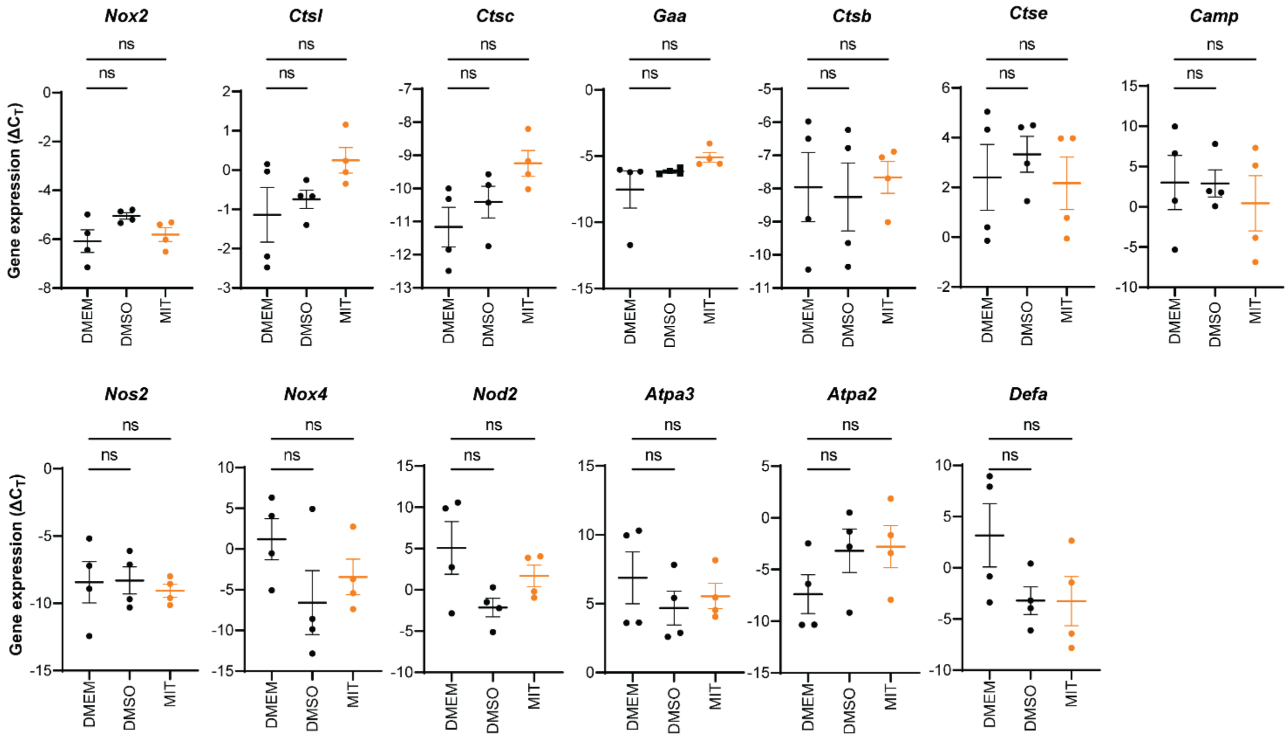

Fig. S12- Lysosomal genes expression not affected by MTX treatment. qRT-PCR analysis of lysosomal genes transcript levels (ΔC_T) in RAW264.7 cells with or without DMSO or MTX treatment overnight. Each dot represents one biological replicate. Statistical analysis was performed using ordinary one-way ANOVA, followed by Tukey's multiple comparison test, NS p > 0.05.

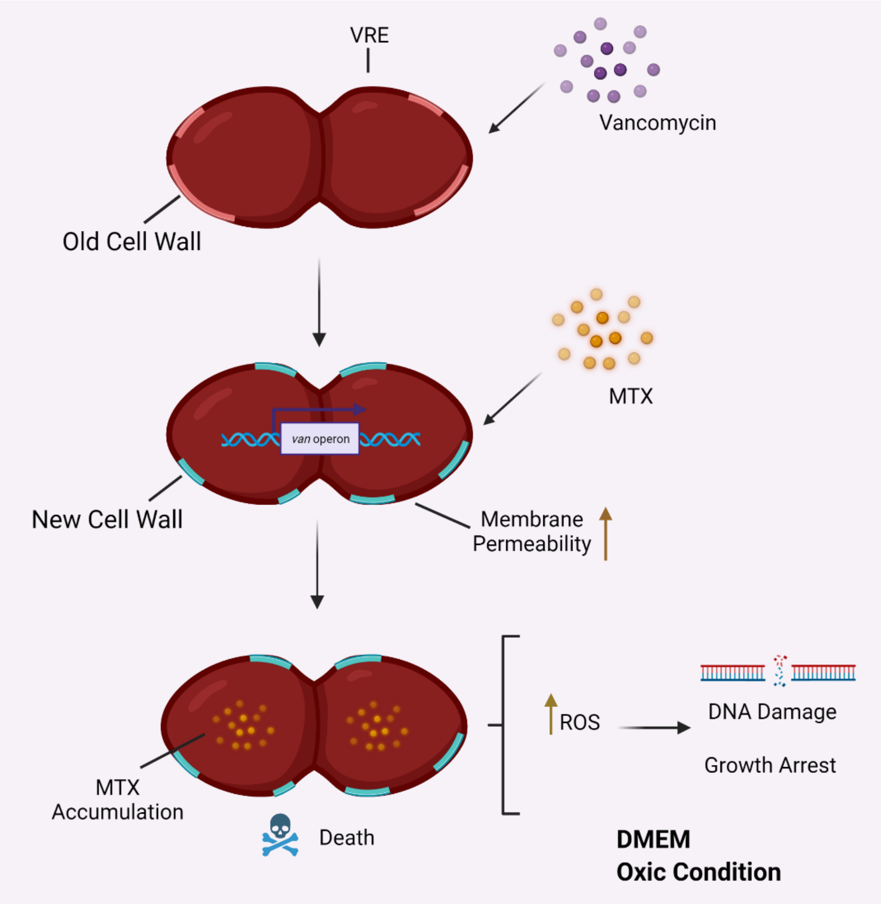

Fig. S13. Proposed mechanism of action for MTX-vancomycin synergy in bacteria in nutrient limiting (DMEM) and oxic conditions.

Vancomycin induced resistance mechanism causes cell wall remodelling and increased permeability, which in turn facilitates MTX uptake. MTX promotes ROS induction, leading to DNA damage and growth arrest.

Table S1 – List of compounds used in initial screen.

Abbreviation	Name	Brand
PEN	Penfluridol	Sigma-Aldrich
TAX	Taxol / Paclitaxel	Sigma-Aldrich
MTX	Mitoxantrone HCL	Sigma-Aldrich
THI	Thiostrepton	Sigma-Aldrich
EVO	Evodiamine	Sigma-Aldrich
CUC	Cucurbitacin I	Sigma-Aldrich
NVP	NVP 231	Sigma-Aldrich
MS	MS 275	SingLab
SMER	SMER3	SingLab
DPI	Diphenyleneiodonium Chloride	SingLab
TRI	Triptolide	Sigma-Aldrich
JTE	JTE 3	Sigma-Aldrich
BOS	Bosutinib	Sigma-Aldrich
QS	QS 11	SingLab
AXI	Axitinib	Sigma-Aldrich
DAS	Dasatinib	Sigma-Aldrich
ARC	Arcyriaflavin A	SingLab
PUR	Purmorphamine	Sigma-Aldrich

Table S2- Antibiotic MIC alone or in the presence of a sub-inhibitory concentration of MTX (0.515 µg/mL).

Antibiotic	MIC (µg/mL)	MIC in combination with MTX (0.515 µg/mL)	Fold Change
Vancomycin	18	0.125	144
Ceftriaxone	300	37.5	8

Daptomycin	0.06	0.007	8
Ampicillin	3	3	-
Rifampicin	0.585	0.585	-
Ciprofloxacin	0.156	0.039	4
Erythromycin	50	50	-
Chloramphenicol	10	2.5	4
Tunicamycin	25	25	-
Gentamicin	500	500	-
Penicillin G	0.5	0.25	2

Table S3- Evolution experiment strain information. Parental and VRE MTX^R strains MIC's are shown.

Strain	Mutation	Annotation and position	Gene	Locus Tag	Vancomycin MIC (μg/mL)	Vancomycin MIC + MTX (0.515 μg/mL):	MTX MIC
***E. faecalis* V583**	Parental				**18.00**	**0.125**	**1.615**
***E. faecalis* V583 MTX^R**	**1:** G→A	G389R (GGG→AGG)	DEAD/DEAH box helicase	EF_RS01660	**18.00**	**12.50**	**20**
	**2:** G→A	intergenic (-54/+67)	Sugar O-acetyltransferase	EF_RS05155			
	**3:** 2 bp→AC	insertion (805-806/912 nt)	Ethanolamine ammonia-lyase subunit EutC	EF_RS07835			
	**4:** (C)5→4	insertion (171/390 nt)	Conjugal transfer protein	EF_RS09020			

Table S4. Strains used in this study

Strain	Reference
E. faecalis OG1RF	*(53)*
E. faecalis OG1RF + pGCP123::VREDEAD	This study
E. faecalis OG1RF + pGCP123::VREMTX^{R}DEAD	This study

E. faecalis OG1RF + pGCP123::VREEF_RS05155	This study
E. faecalis OG1RF + pGCP123::VREMTX^{R}EF_RS05155	This study
E. faecalis OG1RF + pGCP123::VREEF_RS07835	This study
E. faecalis OG1RF + pGCP123::VREMTX^{R}EF_RS07835	This study
E. faecalis OG1RF + pGCP123::VREEF_RS09020	This study
E. faecalis OG1RF + pGCP123::VREMTX^{R}EF_RS09020	This study
E. faecalis OG1RF + pMSP3535::VRE*van*operon	This study
E. faecalis V583	*(54)*
E. faecalis V583 MTX^R	This study
E. coli EC958	*(55)*
E. coli UTI89	*(56)*
S. aureus USA300	*(57)*
S. *typhimurium* 14028S	*(58)*
P. aeruginosa PAO1	*(59)*
E. faecium E745	*(60)*
E. faecium AUS0004	*(61)*
E. faecium E8202	*(62)*
E. faecalis 12030	*(63)*
E. faecalis TTSHW-EF43	*(64)*
E. faecalis ATCC 29121	*(65)*
S. aureus 8325-4	*(66)*
S. aureus MN8	*(67)*
S. aureus 15981	*(68)*
S. aureus ISP479	*(69)*
S. aureus Newman	*(70)*

Table S5. Plasmids used in this study

Plasmid	Plasmid Name	Reference
1	pGCP123	*(49)*
2	pMSP3535	*(70)*, gift from Gary Dunny (Addgene 46886)

Table S6. Primers used in this study

Primer	Primer Name	Sequence (5'-3')
1	DEAD_123_F	GAGGGAGAATCTCGATTGAAAGCAACGTTACATCCCT
2	DEAD_123_R	TACCGTCGACCTCGATTATCCAAGTTTATCCCCCCT
3	35XhoI_Van_F	ACGCCTCGAG ATACTAAGTAATTTGTAG
4	35BamHI_Van_R	ACTGGGATCCAATGATTTTGACCGACAG
5	Vector123_F	GATCCACTAGTTCTAGAGCGGCC
6	Vector123_R	CCCCGGGCTGCAGGAATTC
7	EF_RS05155_F	TCCTGCAGCCCGGGGCTTAGATAAAGGATAGATAAGCAAG
8	EF_RS05155_R	TAGAACTAGTGGATCTTTTATTCATGAAAGAATTGCTTC
9	Vector123_ EF_RS07835_F	AGCGGCCGCCACCGCGGTG
10	Vector123_ EF_RS07835_R	GGGCTGCAGGAATTCGATATCAAG
11	EF_RS07835_F	GAATTCCTGCAGCCCCAATGATAATTCTTTTGCTAATGCC
12	EF_RS07835_R	GCGGTGGCGGCCGCTAAGAATGGATGGAAAAAATGGGC
13	EF_RS09020_F	TCCTGCAGCCCGGGGATGAAAAAAATAAAAAGCTACACCA
14	EF_RS09020_R	TAGAACTAGTGGATCTTAATCGGGTACATAGTTCACACTC
15	sodA_F	TGGGCTTGGTTAGTTGTGAATA
16	sodA_R	TTCCCAAACGTCAAGACCTAAA
17	recA_F	AGATTGCCGATGCCTTAGTT
18	recA_R	CTCCCATCTCACCATCAATCTC
19	lexA_F	CGGAATTCAGCCGACAACTA
20	lexA_R	ATCAGGAGGTAACGGGAAGA
21	groES_F	GTGAAGGTCGTGTGCTTGA
22	groES_R	GGCAATAATGTCTTTGGCTGATAC
23	rpoB_F	CCTGTACCTACCCACGGAGA
24	rpoB_R	TGGACGTTTCACCAAAACAA
25	GAPDH_F	CTAGACCACAGTCCATGCCAT
26	GAPDH_R	ACACATTGGGGGTAGGAACACG
27	CtsD_F	TGAAGACTCCCGGCGTCTT
28	CtsD_R	AATCGCGAAGGAGGACGAA

29	CtsB_F	TGATGCACGGGAACAATGG
30	CtsB_R	GCAGGAGCCCTGGTCTCTAA
31	NOD2_F	GCTGTCTTGGGATGTGCT
32	NOD2_R	GGATGAAGGGAGTGAGTGTC
33	CatL_F	ATGGCACGAATGAGGAAGAG
34	CatL_R	GAAAAAGCCTCCCCTTCTTG
35	Nox2_F	ACTCCTTGGAGCACTGG
36	Nox2_R	GTTCCTGTCCAGTTGTCTTCG
37	Nox4_F	TGAACTACAGTGAAGATTTCCTTGAAC
38	Nox4_R	GACACCCGTCAGACCAGGAA
39	vATPase_A3_F	CACCCGGGGGCCACATTCAG
40	vATPase_A3_R	CCCTCGCGGCACACCAGACC
41	Nos2_F	TTACGTCCATCGTGGACAGC
42	Nos2_R	TGGGCTGGGTGTTAGTCTTA
43	Cramp_F	AAGGAACAGGGGGTGGTG
44	Cramp_R	CCGGGAAATTTTCTTGAACC
45	CtsE_F	CAGTCCGACACATACACG
46	CtsE_R	TGCCCTGGCTCCTTGAC
47	CtsC_F	ACCTGGGTGTTCCAGGTGGGCCC
48	CtsC_R	GCCCGGAATTGCCCAGCTCGTCG
49	HexB_F	GCTGTTGGTGAGAGACTCTGGA
50	HexB_R	GAGGTTGTGCAGCTATTCCACG
51	CstH_F	GCCACCAAAAGTAACTACCTCCG
52	CstH_R	CGTGGTAGAGAAAGTCCAGCAG
53	DefA_F	AACTGAGGAGCAGCCAGGAGAA
54	DefA_R	CTTCCTTTGCAGCCTCTTGATCT
55	GAA_F	ACCGTCCAACTTCGTTAGAGGC
56	GAA_R	ATTGGTGGCTGGAGGCACAGAT

REFERENCES AND NOTES

1. C. J. Murray, K. S. Ikuta, F. Sharara, L. Swetschinski, G. Robles Aguilar, A. Gray, C. Han, C. Bisignano, P. Rao, E. Wool, S. C. Johnson, A. J. Browne, M. G. Chipeta, F. Fell, S. Hackett, G. Haines-Woodhouse, B. H. Kashef Hamadani, E. A. P. Kumaran, B. McManigal, R. Agarwal, S. Akech, S. Albertson, J. Amuasi, J. Andrews, A. Aravkin, E. Ashley, F. Bailey, S. Baker, B. Basnyat, A. Bekker, R. Bender, A. Bethou, J. Bielicki, S. Boonkasidecha, J. Bukosia, C. Carvalheiro, C. Castañeda-Orjuela, V. Chansamouth, S. Chaurasia, S. Chiurchiù, F. Chowdhury, A. J. Cook, B. Cooper, T. R. Cressey, E. Criollo-Mora, M. Cunningham, S. Darboe, N. P. J. Day, M. de Luca, K. Dokova, A. Dramowski, S. J. Dunachie, T. Eckmanns, D. Eibach, A. Emami, N. Feasey, N. Fisher-Pearson, K. Forrest, D. Garrett, P. Gastmeier, A. Z. Giref, R. C. Greer, V. Gupta, S. Haller, A. Haselbeck, S. I. Hay, M. Holm, S. Hopkins, K. C. Iregbu, J. Jacobs, D. Jarovsky, F. Javanmardi, M. Khorana, N. Kissoon, E. Kobeissi, T. Kostyanev, F. Krapp, R. Krumkamp, A. Kumar, H. H. Kyu, C. Lim, D. Limmathurotsakul, M. J. Loftus, M. Lunn, J. Ma, N. Mturi, T. Munera-Huertas, P. Musicha, M. M. Mussi-Pinhata, T. Nakamura, R. Nanavati, S. Nangia, P. Newton, C. Ngoun, A. Novotney, D. Nwakanma, C. W. Obiero, A. Olivas-Martinez, P. Olliaro, E. Ooko, E. Ortiz-Brizuela, A. Y. Peleg, C. Perrone, N. Plakkal, A. Ponce-de-Leon, M. Raad, T. Ramdin, A. Riddell, T. Roberts, J. V. Robotham, A. Roca, K. E. Rudd, N. Russell, J. Schnall, J. A. G. Scott, M. Shivamallappa, J. Sifuentes-Osornio, N. Steenkeste, A. J. Stewardson, T. Stoeva, N. Tasak, A. Thaiprakong, G. Thwaites, C. Turner, P. Turner, H. R. van Doorn, S. Velaphi, A. Vongpradith, H. Vu, T. Walsh, S. Waner, T. Wangrangsimakul, T. Wozniak, P. Zheng, B. Sartorius, A. D. Lopez, A. Stergachis, C. Moore, C. Dolecek, M. Naghavi, Global burden of bacterial antimicrobial resistance in 2019: A systematic analysis. *Lancet* **399**, 629–655 (2022).

2. World Health Organization, New report calls for urgent action to avert antimicrobial resistance crisis; www.who.int/news/item/29-04-2019-new-report-calls-for-urgent-action-to-avert-antimicrobial-resistance-crisis.

3. C. Y. Chiang, I. Uzoma, R. T. Moore, M. Gilbert, A. J. Duplantier, R. G. Panchal, Mitigating the impact of antibacterial drug resistance through host-directed therapies: Current progress, outlook, and challenges. *mBio* **9**, e01932 (2018).

4. A. Giacometti, O. Cirioni, A. M. Schimizzi, M. S. del Prete, F. Barchiesi, M. M. D'Errico, E. Petrelli, G. Scalise, Epidemiology and microbiology of surgical wound infections. *J. Clin. Microbiol.* **38**, 918–922 (2000).

5. P. G. Bowler, B. I. Duerden, D. G. Armstrong, Wound microbiology and associated approaches to wound management. *Clin. Microbiol. Rev.* **14**, 244–269 (2001).

6. S. E. Dowd, Y. Sun, P. R. Secor, D. D. Rhoads, B. M. Wolcott, G. A. James, R. D. Wolcott, Survey of bacterial diversity in chronic wounds using Pyrosequencing, DGGE, and full ribosome shotgun sequencing. *BMC Microbiol.* **8**, 43 (2008).

7. L. J. Bessa, P. Fazii, M. di Giulio, L. Cellini, Bacterial isolates from infected wounds and their antibiotic susceptibility pattern: Some remarks about wound infection. *Int. Wound J.* **12**, 47–52 (2015).

8. M. O. Ahmed, K. E. Baptiste, Vancomycin-resistant enterococci: A review of antimicrobial resistance mechanisms and perspectives of human and animal health. *Microb. Drug Resist.* **24**, 590–606 (2018).

9. Centers for Disease Control and Prevention, 2019 antibiotic resistance threats report; www.cdc.gov/drugresistance/biggest-threats.html#van.

10. H. S. Gold, Vancomycin-resistant enterococci: Mechanisms and clinical observations. *Clin. Infect. Dis.* **33**, 210–219 (2001).

11. P. J. Stogios, A. Savchenko, Molecular mechanisms of vancomycin resistance. *Protein Sci.* **29**, 654–669 (2020).

12. M. L. Faron, N. A. Ledeboer, B. W. Buchan, Resistance mechanisms, epidemiology, and approaches to screening for vancomycin-resistant Enterococcus in the health care setting. *J. Clin. Microbiol.* **54**, 2436–2447 (2016).

13. B. Y. Q. Tien, H. M. S. Goh, K. K. L. Chong, S. Bhaduri-Tagore, S. Holec, R. Dress, F. Ginhoux, M. A. Ingersoll, R. B. H. Williams, K. A. Kline, *Enterococcus faecalis* promotes innate immune

suppression and polymicrobial catheter-associated urinary tract infection. *Infect. Immun.* **85**, e00378-17 (2017).

14. R. A. G. da Silva, W. H. Tay, F. K. Ho, F. R. Tanoto, K. K. L. Chong, P. Y. Choo, A. Ludwig, K. A. Kline, *Enterococcus faecalis* alters endo-lysosomal trafficking to replicate and persist within mammalian cells. *PLOS Pathog.* **18**, e1010434 (2022).

15. K. K. L. Chong, W. H. Tay, B. Janela, A. M. H. Yong, T. H. Liew, L. Madden, D. Keogh, T. M. S. Barkham, F. Ginhoux, D. L. Becker, K. A. Kline, *Enterococcus faecalis* modulates immune activation and slows healing during wound infection. *J. Infect. Dis.* **216**, 1644–1654 (2017).

16. F. C. Fang, Antimicrobial reactive oxygen and nitrogen species: Concepts and controversies. *Nat. Rev. Microbiol.* **2**, 820–832 (2004).

17. J. M. Slauch, How does the oxidative burst of macrophages kill bacteria? Still an open question. *Mol. Microbiol.* **80**, 580–583 (2011).

18. J. R. Sheldon, E. P. Skaar, Metals as phagocyte antimicrobial effectors. *Curr. Opin. Immunol.* **60**, 1–9 (2019).

19. G. L. Lukacs, O. D. Rotstein, S. Grinstein, Phagosomal acidification is mediated by a vacuolar-type H^+-ATPase in murine macrophages. *J. Biol. Chem.* **265**, 21099–21107 (1990).

20. E. Uribe-Querol, C. Rosales, Phagocytosis: Our current understanding of a universal biological process. *Front. Immunol.* **11**, 1066 (2020).

21. M. Orecchioni, Y. Ghosheh, A. B. Pramod, K. Ley, Macrophage polarization: Different gene signatures in M1(Lps+) vs. Classically and M2(LPS-) vs. Alternatively activated macrophages. *Front. Immunol.* **10**, 1084 (2019).

22. P. J. Murray, Macrophage polarization.*Annu. Rev. Physiol.* **79**, 541–566 (2017).

23. C. D. Mills, K. Kincaid, J. M. Alt, M. J. Heilman, A. M. Hill, M-1/M-2 macrophages and the Th1/Th2 paradigm. *J. Immunol.* **164**, 6166–6173 (2000).

24. U. Theuretzbacher, K. Outterson, A. Engel, A. Karlén, The global preclinical antibacterial pipeline. *Nat. Rev. Microbiol.* **18**, 275–285 (2020).

25. G. Hu, Y. Su, B. H. Kang, Z. Fan, T. Dong, D. R. Brown, J. Cheah, K. D. Wittrup, J. Chen, High-throughput phenotypic screen and transcriptional analysis identify new compounds and targets for macrophage reprogramming. *Nat. Commun.* **12**, 733 (2021).

26. M. J. Rybak, B. M. Lomaestro, J. C. Rotschafer, R. C. Moellering, W. A. Craig, M. Billeter, J. R. Dalovisio, D. P. Levine, Vancomycin therapeutic guidelines: A summary of consensus recommendations from the infectious diseases society of America, the American society of health-system pharmacists, and the society of infectious diseases pharmacists. *Clin. Infect. Dis.* **49**, 325–327 (2009).

27. R. F. Novak, E. D. Kharasch, Mitoxantrone: Propensity for free radical formation and lipid peroxidation—Implications for cardiotoxicity. *Invest. New Drugs* **3**, 95–99 (1985).

28. F. Baquero, B. R. Levin, Proximate and ultimate causes of the bactericidal action of antibiotics. *Nat. Rev. Microbiol.* **19**, 123–132 (2020).

29. C. Watanakunakorn, Mode of action and in-vitro activity of vancomycin. *J. Antimicrob. Chemother.* **14** (Suppl D), 7–18 (1984).

30. D. H. Bell, Characterization of the fluorescence of the antitumor agent, mitoxantrone. *Biochim. Biophys. Acta* **949**, 132–137 (1988).

31. P. Redder, S. Hausmann, V. Khemici, H. Yasrebi, P. Linder, Bacterial versatility requires DEAD-box RNA helicases. *FEMS Microbiol. Rev.* **39**, 392–412 (2015).

32. M. Benoit, B. Desnues, J.-L. Mege, Macrophage polarization in bacterial infections. *J. Immunol.* **181**, 3733–3739 (2008).

33. Q. Huang, J. Hou, P. Yang, J. Yan, X. Yu, Y. Zhuo, S. He, F. Xu, Antiviral activity of mitoxantrone dihydrochloride against human herpes simplex virus mediated by suppression of the viral immediate early genes. *BMC Microbiol.* **19**, 274 (2019).

34. P. F. Chan, V. Srikannathasan, J. Huang, H. Cui, A. P. Fosberry, M. Gu, M. M. Hann, M. Hibbs, P. Homes, K. Ingraham, J. Pizzollo, C. Shen, A. J. Shillings, C. E. Spitzfaden, R. Tanner, A. J. Theobald, R. A. Stavenger, B. D. Bax, M. N. Gwynn, Structural basis of DNA gyrase inhibition by antibacterial QPT-1, anticancer drug etoposide and moxifloxacin. *Nat. Commun.* **6**, 10048 (2015).

35. E. J. Fox, Mechanism of action of mitoxantrone. *Neurology* **63**, S15–S18 (2004).

36. R. J. Worthington, C. Melander, Combination approaches to combat multi-drug resistant bacteria. *Trends Biotechnol.* **31**, 177–184 (2013).

37. K. J. I. Thorne, R. C. Oliver, A. J. Barrett, Lysis and killing of bacteria by lysosomal proteinases. *Infect. Immun.* **14**, 555–563 (1976).

38. A. Reis-mendes, J. L. Dores-sousa, A. I. Padrão, M. Duarte-araújo, J. A. Duarte, V. Seabra, S. Gonçalves-monteiro, F. Remião, F. Carvalho, E. Sousa, M. L. Bastos, V. M. Costa, Inflammation as a possible trigger for mitoxantrone-induced cardiotoxicity: An in vivo study in adult and infant mice. *Pharmaceuticals* **14**, 510 (2021).

39. L. Fischer-Riepe, N. Daber, J. Schulte-Schrepping, B. C. Véras De Carvalho, A. Russo, M. Pohlen, J. Fischer, A. I. Chasan, M. Wolf, T. Ulas, S. Glander, C. Schulz, B. Skryabin, A. Wollbrink Dipl-Ing, N. Steingraeber, C. Stremmel, M. Koehle, F. Gärtner, S. Vettorazzi, D. Holzinger, J. Gross, F. Rosenbauer, M. Stoll, S. Niemann, J. Tuckermann, J. L. Schultze, J. Roth, K. Barczyk-Kahlert, CD163 expression defines specific, IRF8-dependent, immune-modulatory macrophages in the bone marrow. *J. Allergy Clin. Immunol.* **146**, 1137–1151 (2020).

40. D. Parker, CD80/CD86 signaling contributes to the proinflammatory response of Staphylococcus aureus in the airway. *Cytokine* **107**, 130–136 (2018).

41. M. C. Marchitto, C. A. Dillen, H. Liu, R. J. Miller, N. K. Archer, R. V. Ortines, M. P. Alphonse, A. I. Marusina, A. A. Merleev, Y. Wang, B. L. Pinsker, A. S. Byrd, I. D. Brown, A. Ravipati, E. Zhang, S. S. Cai, N. Limjunyawong, X. Dong, M. R. Yeaman, S. I. Simon, W. Shen, S. K. Durum, R. L. O'Brien, E. Maverakis, L. S. Miller, Clonal $V\gamma6^{+}V\delta4^{+}$ T cells promote IL-17–mediated immunity

against *Staphylococcus aureus* skin infection. *Proc. Natl. Acad. Sci. U.S.A.* **166**, 10917–10926 (2019).

42. C. A. Dillen, B. L. Pinsker, A. I. Marusina, A. A. Merleev, O. N. Farber, H. Liu, N. K. Archer, D. B. Lee, Y. Wang, R. V. Ortines, S. K. Lee, M. C. Marchitto, S. S. Cai, A. G. Ashbaugh, L. S. May, S. M. Holland, A. F. Freeman, L. G. Miller, M. R. Yeaman, S. I. Simon, J. D. Milner, E. Maverakis, L. S. Miller, Clonally expanded γδ T cells protect against *Staphylococcus aureus* skin reinfection. *J. Clin. Invest.* **128**, 1026–1042 (2018).

43. L. J. Juttukonda, W. N. Beavers, D. Unsihuay, K. Kim, G. Pishchany, K. J. Horning, A. Weiss, H. Al-Tameemi, J. M. Boyd, G. A. Sulikowski, A. B. Bowman, E. P. Skaara, A small-molecule modulator of metal homeostasis in gram-positive pathogens. *mBio* **11**, e02555-20 (2020).

44. L. Boulos, M. Prévost, B. Barbeau, J. Coallier, R. Desjardins, LIVE/DEAD® BacLight™: Application of a new rapid staining method for direct enumeration of viable and total bacteria in drinking water. *J. Microbiol. Methods* **37**, 77–86 (1999).

45. L. Cui, Y. H. Lee, T. L. Thein, J. Fang, J. Pang, E. E. Ooi, Y. S. Leo, C. N. Ong, S. R. Tannenbaum, Serum metabolomics reveals serotonin as a predictor of severe dengue in the early phase of dengue fever. *PLOS Negl. Trop. Dis.* **10**, e0004607 (2016).

46. B. Luna, V. Trebosc, B. Lee, M. Bakowski, A. Ulhaq, J. Yan, P. Lu, J. Cheng, T. Nielsen, J. Lim, W. Ketphan, H. Eoh, C. McNamara, N. Skandalis, R. She, C. Kemmer, S. Lociuro, G. E. Dale, B. Spellberg, A nutrient-limited screen unmasks rifabutin hyperactivity for extensively drug-resistant Acinetobacter baumannii. *Nat. Microbiol.* **5**, 1134–1143 (2020).

47. K. L. Palmer, A. Daniel, C. Hardy, J. Silverman, M. S. Gilmore, Genetic basis for daptomycin resistance in enterococci. *Antimicrob. Agents Chemother.* **55**, 3345–3356 (2011).

48. H. V. Nielsen, P. S. Guiton, K. A. Kline, G. C. Port, J. S. Pinkner, F. Neiers, S. Normark, B. Henriques-Normark, M. G. Caparon, S. J. Hultgren, The metal ion-dependent adhesion site motif of the *Enterococcus faecalis* EbpA pilin mediates pilus function in catheter-associated urinary tract infection. *mBio* **3**, e00177 (2012).

49. S. Manzanero, Generation of mouse bone marrow-derived macrophages. *Methods Mol. Biol.* **844**, 177–181 (2012).

50. K. J. Livak, T. D. Schmittgen, Analysis of relative gene expression data using real-time quantitative PCR and the $2^{-\Delta\Delta C}{}_{T}$ method. *Methods* **25**, 402–408 (2001).

51. K. Eliceiri, C. A. Schneider, W. S. Rasband, K. W. Eliceiri, NIH Image to ImageJ: 25 years of image analysis. *Nat. Methods* **9**, 671–675 (2012).

52. G. M. Dunny, B. L. Brown, D. B. Clewell, Induced cell aggregation and mating in Streptococcus faecalis: Evidence for a bacterial sex pheromone. *Proc. Natl. Acad. Sci. U.S.A.* **75**, 3479–3483 (1978).

53. A. Bourgogne, D. A. Garsin, X. Qin, K. V. Singh, J. Sillanpaa, S. Yerrapragada, Y. Ding, S. Dugan-Rocha, C. Buhay, H. Shen, G. Chen, G. Williams, D. Muzny, A. Maadani, K. A. Fox, J. Gioia, L. Chen, Y. Shang, C. A. Arias, S. R. Nallapareddy, M. Zhao, V. P. Prakash, S. Chowdhury, H. Jiang, R. A. Gibbs, B. E. Murray, S. K. Highlander, G. M. Weinstock, Large scale variation in *Enterococcus faecalis* illustrated by the genome analysis of strain OG1RF. *Genome Biol.* **9**, R110 (2008).

54. M. Totsika, S. A. Beatson, S. Sarkar, M. D. Phan, N. K. Petty, N. Bachmann, M. Szubert, H. E. Sidjabat, D. L. Paterson, M. Upton, M. A. Schembri, Insights into a multidrug resistant Escherichia coli pathogen of the globally disseminated ST131 lineage: Genome analysis and virulence mechanisms. *PLOS ONE* **6**, e26578 (2011).

55. S. L. Chen, C. S. Hung, J. Xu, C. S. Reigstad, V. Magrini, A. Sabo, D. Blasiar, T. Bieri, R. R. Meyer, P. Ozersky, J. R. Armstrong, R. S. Fulton, J. P. Latreille, J. Spieth, T. M. Hooton, E. R. Mardis, S. J. Hultgren, J. I. Gordon, Identification of genes subject to positive selection in uropathogenic strains of Escherichia coli: A comparative genomics approach. *Proc. Natl. Acad. Sci. U.S.A.* **103**, 5977–5982 (2006).

56. L. K. McDougal, C. D. Steward, G. E. Killgore, J. M. Chaitram, S. K. McAllister, F. C. Tenover, Pulsed-field gel electrophoresis typing of oxacillin-resistant Staphylococcus aureus isolates from the United States: Establishing a national database. *J. Clin. Microbiol.* **41**, 5113–5120 (2003).

57. Judicial Commission of the International Committee on Systematics of Prokaryotes, *The type species of the genus Salmonella Lignieres 1900 is Salmonella enterica (ex Kauffmann and Edwards 1952) Le Minor and Popoff 1987, with the type strain LT2T, and conservation of the epithet enterica in Salmonella enterica over all earlier epithets that may be applied to this species. Opinion 80. Int. J. Syst. Evol. Microbiol.* **55**, 519–520 (2005).

58. M. Hentzer, K. Riedel, T. B. Rasmussen, A. Heydorn, J. B. Andersen, M. R. Parsek, S. A. Rice, L. Eberl, S. Molin, N. Høiby, S. Kjelleberg, M. Givskov, Inhibition of quorum sensing in Pseudomonas aeruginosa biofilm bacteria by a halogenated furanone compound. *Microbiology* **148**, 87–102 (2002).

59. X. Zhang, V. de Maat, A. M. Guzmán Prieto, T. K. Prajsnar, J. R. Bayjanov, M. de Been, M. R. C. Rogers, M. J. M. Bonten, S. Mesnage, R. J. L. Willems, W. van Schaik, RNA-seq and Tn-seq reveal fitness determinants of vancomycin-resistant Enterococcus faecium during growth in human serum. *BMC Genomics* **18**, 893 (2017).

60. M. M. C. Lam, T. Seemann, D. M. Bulach, S. L. Gladman, H. Chen, V. Haring, R. J. Moore, S. Ballard, M. L. Grayson, P. D. R. Johnson, B. P. Howden, T. P. Stineara, Comparative analysis of the first complete *Enterococcus faecium* genome. *J. Bacteriol.* **194**, 2334–2341 (2012).

61. J. Top, S. Arredondo-Alonso, A. C. Schürch, S. Puranen, M. Pesonen, J. Pensar, R. J. L. Willems, J. Corander, Genomic rearrangements uncovered by genome-wide co-evolution analysis of a major nosocomial pathogen, *Enterococcus faecium*. *Microb. Genom.* **6**, mgen000488 (2020).

62. Y. Wang, J. Huebner, A. O. Tzianabos, G. Martirosian, D. L. Kasper, G. B. Pier, Structure of an antigenic teichoic acid shared by clinical isolates of Enterococcus faecalis and vancomycin-resistant Enterococcus faecium. *Carbohydr. Res.* **316**, 155–160 (1999).

63. J. H. Ch'ng, M. Muthu, K. K. L. Chong, J. J. Wong, C. A. Z. Tan, Z. J. S. Koh, D. Lopez, A. Matysik, Z. J. Nair, T. Barkham, Y. Wang, K. A. Kline, Heme cross-feeding can augment *Staphylococcus aureus* and *Enterococcus faecalis* dual species biofilms. *ISME J.* **16**, 2015–2026 (2022).

64. ATCC, Enterococcus faecalis (Andrewes and Horder) Schleifer and Kilpper-Balz 29212; www.atcc.org/products/29212.

65. A. J. O'Neill, *Staphylococcus aureus* SH1000 and 8325-4: Comparative genome sequences of key laboratory strains in staphylococcal research. *Lett. Appl. Microbiol.* **51**, 358–361 (2010).

66. D. A. Blomster-Hautamaa, P. M. Schlievert, Preparation of toxic shock syndrome toxin-1. *Methods Enzymol.* **165**, 37–43 (1988).

67. J. Valle, A. Toledo-Arana, C. Berasain, J. M. Ghigo, B. Amorena, J. R. Penadés, I. Lasa, SarA and not σ^B is essential for biofilm development by *Staphylococcus aureus*. *Mol. Microbiol.* **48**, 1075–1087 (2003).

68. A. T. Giraudo, C. G. Raspanti, A. Calzolari, R. Nagel, Characterization of a Tn551-mutant of Staphylococcus aureus defective in the production of several exoproteins. *Can. J. Microbiol.* **40**, 677–681 (1994).

69. D. McDevitt, P. Vaudaux, T. J. Foster, Genetic evidence that bound coagulase of *Staphylococcus aureus* is not clumping factor. *Infect. Immun.* **60**, 1514–1523 (1992).

70. E. M. Bryan, T. Bae, M. Kleerebezem, G. M. Dunny, Improved vectors for nisin-controlled expression in gram-positive bacteria. *Plasmid* **44**, 183–190 (2000).
